# Alpha and Theta Oscillations Associated With Behavioral Phenotypes of Pain–Attention Interaction

**DOI:** 10.1002/brb3.70190

**Published:** 2025-01-19

**Authors:** Nikou Kelardashti, Benjamin T. Dunkley, Rima El‐Sayed, Vaidhehi Veena Sanmugananthan, Junseok Andrew Kim, Natalie Rae Osborne, Joshua C. Cheng, Anton Rogachov, Rachael L. Bosma, Ariana E. Besik, Karen Deborah Davis

**Affiliations:** ^1^ Division of Brain, Imaging and Behavior, Krembil Brain Institute, Krembil Research Institute University Health Network Toronto Ontario Canada; ^2^ Institute of Medical Science University of Toronto Toronto Ontario Canada; ^3^ Department of Diagnostic Imaging Hospital for Sick Children Toronto Ontario Canada; ^4^ Neurosciences & Mental Health SickKids Research Institute Toronto Ontario Canada; ^5^ Department of Medical Imaging University of Toronto Toronto Ontario Canada; ^6^ Department of Psychology University of Nottingham Nottingham UK; ^7^ Department of Surgery University of Toronto Toronto Ontario Canada

**Keywords:** alpha oscillations, attention, default mode network, pain, salience network, somatosensory cortex, theta oscillations

## Abstract

**Purpose:**

Pain is inherently salient and so draws our attention in addition to impacting performance on attention‐demanding tasks. Individual variability in pain–attention interactions can be assessed by two kinds of behavioral phenotypes that quantify how individuals prioritize pain versus attentional needs. The intrinsic attention to pain (IAP) measure quantifies the degree to which a person attends to pain (high‐IAP) or mind‐wanders away from pain (low‐IAP). The A/P categorization quantifies how pain impacts cognitive performance during an attention‐demanding task classifying individuals into P type (pain dominates, worse performance during pain in comparison to no pain) and A type (attention to task dominates, better performance during pain in comparison to no pain). Although previous MRI‐based studies have linked these phenotypes with the dynamic pain connectome (DPC), the underlying neural oscillations are not known. This paper aims to examine the brain–behavior relationship between alpha and theta oscillations within nodes of the DPC and pain–attention phenotypes.

**Method:**

Fifty participants (27 F, 23 M) underwent resting‐state magnetoencephalography (MEG). Individual IAP scores were determined by assessing mind‐wandering during pain and A/P type was based on interference of pain with cognitive task performance.

**Finding:**

The main findings were: (1) peak alpha frequency (PAF) power did not differ between low/high‐IAP individuals or A/P‐type individuals within the nodes of the DPC; (2) compared to high‐IAP individuals, those with low‐IAP have slower PAF in the left primary somatosensory cortex, posterior cingulate cortex and precuneus and higher theta power in the ascending nociceptive pathway and default mode network; (3) males with low‐IAP, compared to females, had higher PAF power throughout the DPC.

**Conclusion:**

Alpha and theta oscillations within the DPC may underlie aspects of attentional focus and pain–attention interactions.

## Graphical abstract

We determined the brain–behavior relationships between resting‐state alpha and theta oscillations within the dynamic pain connectome and pain–attention phenotypes (IAP and A/P). Compared to those with high IAP, individuals with low IAP had higher theta power. However, alpha activity is similar for those who attend to pain versus another focus.

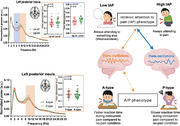

## 1 Introduction

Pain manifests differently in individuals, with affective and cognitive factors impacting the pain experience (Bushnell, Čeko, and Low 2013; Melzack and Casey 1968). Attention is one of the key factors that can influence our experience of pain. However, the interaction between attention and pain and the underlying mechanisms are not fully understood (Legrain et al. 2009; Torta et al. 2017). Two kinds of behavioral phenotypes have been previously delineated based on differences in the degree to which an individual attends to pain and how pain impacts cognitive performance, as an indicator of whether individuals prioritize attending to pain versus task performance. One phenotype classifies individuals based on an intrinsic attention to pain (IAP) score, which quantifies the degree to which they attend to painful stimuli (high‐IAP) versus how much they mind‐wander away from pain (low‐IAP) (Kucyi, Salomons, and Davis 2013). The other type of behavioral phenotype classifies individuals based on how pain impacts cognitive performance during an attention‐demanding task (Cheng et al. 2017; Erpelding and Davis 2013; Seminowicz, Mikulis, and Davis 2004). Individuals are designated as A type (i.e., attention to task is prioritized) when they have better task performance (i.e., faster reaction time) when painful stimuli are applied compared to a pain‐free condition. Conversely, individuals are designated as P type (pain is prioritized) when they exhibit diminished task performance (i.e., slower reaction time) during concurrent pain compared to a no‐pain condition.

Our lab has previously used MRI‐based structural and functional imaging approaches to identify brain–behavioral relationships that distinguish A from P types and high versus low IAP types (Cheng et al. 2017; Erpelding and Davis 2013; Kucyi, Salomons, and Davis 2013; Seminowicz, Mikulis, and Davis 2004). These studies also contributed to the development of the concept of the dynamic pain connectome (DPC) that is a system that includes the ascending nociceptive pathways, descending antinociceptive pathways, default mode network (DMN), and salience network (SN) (Kucyi and Davis 2015). However, the fine temporal dynamics of brain activity are not discernible using hemodynamic‐based fMRI approaches. A deeper understanding of the brain dynamics that underlie pain–attention phenotypes requires a technique with high temporal resolution such as magnetoencephalography (MEG) that can be used to examine resting‐state neural oscillations within the DPC with millisecond precision (Kim and Davis 2021).

Studies of neural oscillations using electroencephalography (EEG) and MEG have shown a relationship between alpha oscillations (8–13 Hz) and acute pain sensitivity as well as prolonged pain (Furman et al. 2018, 2020, 2019; Giehl et al. 2014). Importantly, study of these oscillations in healthy individuals experiencing acute pain can provide insight into the findings in chronic pain populations, showing slowing of the peak alpha frequency (PAF), defined as the frequency in the alpha range (8–13 Hz) that has the greatest power (Fauchon et al. 2022a, 2022b; Kim et al. 2019, 2020; Kisler et al. 2020), and aberrant theta oscillations (4–8 Hz) (Fallon et al. 2018; Sarnthein et al. 2006; Stern, Jeanmonod, and Sarnthein 2006; Walton, Dubois, and Llinas 2010). Notably, both theta and alpha oscillations are known to be involved in attention processes (Clayton et al. 2015; Foxe and Snyder 2011; Klimesch 2012). Recognizing the distinct roles of alpha and theta oscillations in pain and attention, it is important to investigate how these oscillations interact within the context of pain–attention dynamics in healthy individuals to inform the aberrant oscillations seen in people with chronic pain.

Given the recent and growing evidence in the pain field of the importance of alpha oscillations, our original a priori aim was to study brain–behavior relationships of pain–attention phenotypes that focused on alpha oscillations. However, initial inspection of power spectral density graphs revealed some prominent findings in the theta band. Therefore, we expanded the aim of our study to examine the brain–behavior relationships between alpha and theta oscillations within DPC and behavioral pain–attention phenotypes (i.e., IAP and A/P).

## 2 Materials and Methods

### 2.1 Participants

Participants were recruited through posted advertisements at the University Health Network and associated research institutes as well as through word of mouth. This was a retrospective study based on MEG and behavioral data previously collected (Fauchon et al. 2022b; Kim et al. 2019; Kisler et al. 2020; Sanmugananthan et al. 2023) from a total of 50 participants. For the IAP analysis, data from all 50 participants who had both MEG and IAP data were used (age (mean ± SD) = 26.9 ± 4.9; 27 women, 23 men; Table 1) and data from 47 of these participants who had both MEG and A/P data were used for the A/P phenotype (age (mean ± SD) = 26.7 ± 4.8; 25 women, 22 men; Table 1). All participants provided written informed consent to procedures approved by the University Health Network Research Ethics Board. The inclusion criteria for healthy participants were (1) absence of acute pain or a history of chronic pain; (2) no diagnosis of neurologic, psychiatric, or metabolic conditions; (3) no history of major surgery; (4) no medication use on a regular basis; (5) no contraindications for MEG or MRI; (6) right handedness. Furthermore, participants above the age of 40 were excluded to avoid the potential confound of age‐related slowing of alpha oscillations and reduced alpha band power (Babiloni et al. 2006; Grandy et al. 2013; Merkin et al. 2023; Scally et al. 2018; Tröndle et al. 2023).

**TABLE 1:** Demographic information of participants for IAP and A/P phenotypes.

(A) IAP phenotype						
Participant group	Total participants (*N*)	Mean age in years (SD)	Female participants (*N*)	Mean female age in years (SD)	Male participants (*N*)	Mean male age in years (SD)
Total	50	26.9 (4.9)	27	26.9 (4.7)	23	26.8 (5.3)
Low IAP	26	27.6 (5.4)	11	28.4 (5.0)	15	27 (5.8)
High IAP	24	26.1 (4.3)	16	25.9 (4.3)	8	26.5 (4.5)

(B) A/P phenotype						
Participant group	Total participants (*N*)	Mean age in years (SD)	Female participants (*N*)	Mean female age in years (SD)	Male participants (*N*)	Mean male age in years (SD)
Total	47	26.7 (4.8)	25	26.4 (4.5)	22	27.1 (5.2)
A type	35	26.7 (4.9)	18	25.7 (4.2)	17	27.8 (5.5)
P type	12	26.8 (4.5)	7	28.3 (4.7)	5	24.8 (3.8)

*Note*: The table presents the demographic information for the (A) IAP phenotype and (B) A/P phenotype. For each phenotype, number of participants (*N*), mean age in years with standard deviation (SD), and counts for each subtype are presented. Tables incorporate sex‐segregated information, presenting data for women and men separately.

### 2.2 Behavioral Data Acquisition

As previously described (Cheng et al. 2017; Erpelding and Davis 2013; Kucyi, Salomons, and Davis 2013; Sanmugananthan et al. 2023; Seminowicz, Mikulis, and Davis 2004), participants underwent a behavioral session which included paradigms to assess IAP and A/P. For each participant, the stimulus intensity was calibrated to elicit a pain intensity rating between 40 and 60 out of 100 (0 = no pain, 100 = worst pain imaginable). The stimuli were delivered to the left forearm over the median nerve using a transcutaneous electrical nerve stimulation device (TENS; 300‐PV, Empi Inc., MN, USA), with a symmetric waveform at 50 Hz and a pulse width set to 250 µs.

#### 2.2.1 IAP Data Acquisition

**FIGURE 1:**
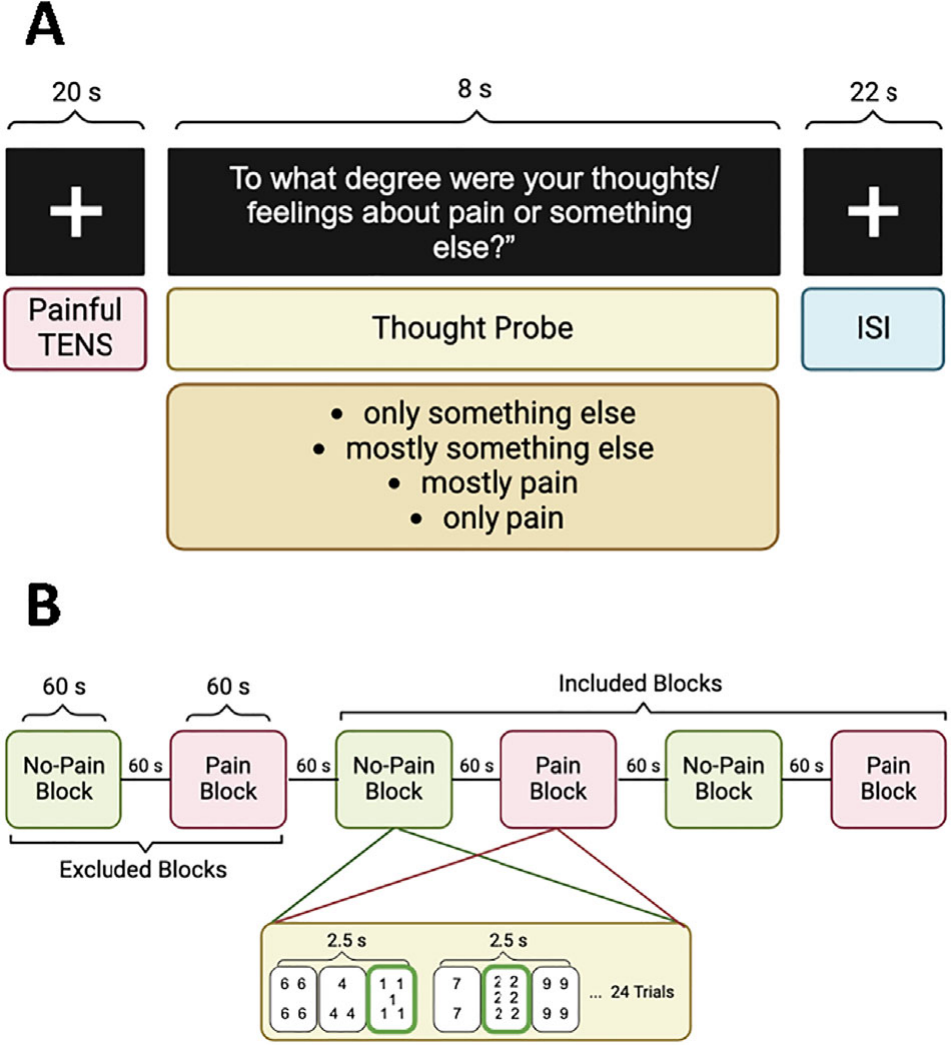
The task trial designs for the (A) experience sampling for IAP phenotype and (B) numeric interference task for A/P phenotype. ISI, interstimulus interval; TENS, transcutaneous electrical nerve stimulation. Figure created with BioRender.com.

An individual's IAP score was quantified using the experience‐sampling task as described previously (Kucyi, Salomons, and Davis 2013). This score quantifies the extent to which individuals remain attentive to their pain. The experience‐sampling task consisted of 20 trials and the structure of each trial is shown in Figure 1A. During each trial, participants were instructed to fixate on a white cross against a black background screen while being exposed to a painful electric stimulus for 20 s. Following the cessation of the stimulus, an 8 s poststimulation thought probe appeared on the screen to evaluate the degree to which participants were directing their attention to the painful stimulus. The thought probe presented on the screen asked: “To what degree were your thoughts/feelings about pain or something else?” Participants were instructed to choose one of the four possible responses of “only pain,” “mostly pain,” “mostly something else,” and “only something else.” A 22 s stimulus‐free interval separated the trials. For each participant, an IAP score was calculated based on the number of trials in which they reported attention toward pain (i.e., “only pain,” “mostly pain”) or attention away from pain (i.e., “only something else,” “mostly something else). This score ranged from −2 to +2, with −2 indicating an individual who consistently responded attention away from pain, and +2 denoting an individual who consistently responded attention toward pain. In addition, for group comparison analyses, the participants were categorized as low‐IAP group (i.e., those with IAP score between −2 and 0) and high‐IAP group (i.e., those with IAP score between 0 and +2). The formula for calculating the IAP score, where *n* is the number of trials, was:

$$\begin{array}{ccc} \mathrm{IAP}\;\mathrm{score}\; & = & \;\left[\left(2n_{\mathrm{onlypain}}+n_{\mathrm{mostlypain}}\right)\;-\;\left(2n_{\mathrm{onlyelse}}+n_{\mathrm{mostlyelse}}\right)\right]/\left(n_{\mathrm{total}}\right) \end{array}$$

#### 2.2.2 A/P Data Acquisition

For the A/P phenotype, we used the numeric interference (NI) task shown in Figure 1B and described previously (Cheng et al. 2017; Erpelding and Davis 2013; Sanmugananthan et al. 2023). This task assessed the impact of an experimental painful stimulus on task performance (reaction time), and categorized participants into A‐ and P‐type groups. In the NI task, participants completed six blocks of trials, with blocks alternating between no‐pain and pain conditions. During pain blocks, participants were required to perform the task while receiving a concurrent noxious electric stimulus. Throughout this task, participants viewed a computer screen that displayed three boxes (i.e., cards), each containing a different number of the same digit between the values of 1–9. Participants had to determine the greatest number of digits that occurred in any of the three boxes and to make a button press to indicate this number as quickly and accurately as possible using a numeric keypad with their right hand. The task is designed to be attention demanding, introducing difficulty through incongruency (Erpelding and Davis 2013; Seminowicz, Mikulis, and Davis 2004). This incongruency required participants to prioritize reporting the nondominant information (i.e., the greatest number of digits) over the dominant information (i.e., highest value). The NI task had six blocks, each lasting 60 s. Within each block, there were 24 trials each taking 2.5 s. Blocks were separated by a 60 s interval and began with an initial no‐pain condition block.

To reduce the impact of learning effects, the first two blocks (no‐pain block and pain block) were excluded from the data analysis. Each individual's performance in the two pain and two no‐pain blocks of the NI task was quantified by calculating the mean reaction time (RT_mean_) for each condition (Cheng et al. 2017; Sanmugananthan et al. 2023; Whelan 2008). Mean reaction time served as a metric of the speed at which individuals completed the task in each condition. Trials with reaction times below 200 ms or exceeding 2500 ms were excluded (Sanmugananthan et al. 2023). The lower limit was set based on an estimate of a minimum time needed for physiological processes during a simple reaction time and the upper limit represents the maximum duration of each trial (Woods et al. 2015).

Individuals were characterized as A or P type based on the difference in mean reaction time (ΔRT_mean_) between the no‐pain and pain blocks. For each individual, the mean reaction time for the no‐pain blocks was subtracted from the mean reaction time for the pain blocks as follows:

$$\Delta {\mathrm{RT}}_{\mathrm{mean}}={\mathrm{RT}}_{\mathrm{mean}\;\mathrm{pain}}\;-{\mathrm{RT}}_{\mathrm{mean}\;\mathrm{no}-\mathrm{pain}}\;$$

Individuals who exhibited a negative ΔRT_mean_, indicating a faster reaction time in pain trials compared to no‐pain trials, were classified as A type. Conversely, individuals who had a positive ΔRT_mean_, signifying a slower reaction time in pain trials compared to no‐pain trials, were categorized as P type.

### 2.3 MEG and MRI Data Acquisition

The MEG data were acquired during a 5‐min resting‐state scan with a 306‐channel Elekta Neuromag TRIUX system. The sampling rate was 1000 Hz with a recording DC bandpass of 330 Hz. Participants were screened to be free of any metallic object or traces of metal from makeup and hair products. Before the scan, fiducial reference points were marked at the nasion, bilateral preauricular positions, and five head‐position coils for the purpose of motion correction and co‐registration with the MRI scan. Participants maintained an upright‐seated position, keeping their eyes open and focusing on a white cross displayed on a black screen in a dark room, while refraining from structured thinking. The tSSS algorithm within the MaxFilter program was used for artefact correction.

Following the resting‐state MEG scan, a high‐resolution T1 anatomical MRI scan was acquired (3T GE scanner, Chicago, IL) in order to co‐register the MEG data using the fiducial points obtained prior to scanning. The co‐registration was for the purpose of source reconstruction. The MRI session included a high resolution T1‐weighted anatomical scan (3D IR‐FSPGR sequence; 180 axial slices; repetition time (TR) = 7.8 ms; echo time (TE) = 3 ms; inversion time (TI) = 450 ms; flip angle = 15°; field‐of‐view (FOV) = 256mm × 256 mm; 256 × 256 matrix; voxel size = 1 mm^3^).

### 2.4 Regions of Interest

**FIGURE 2:**
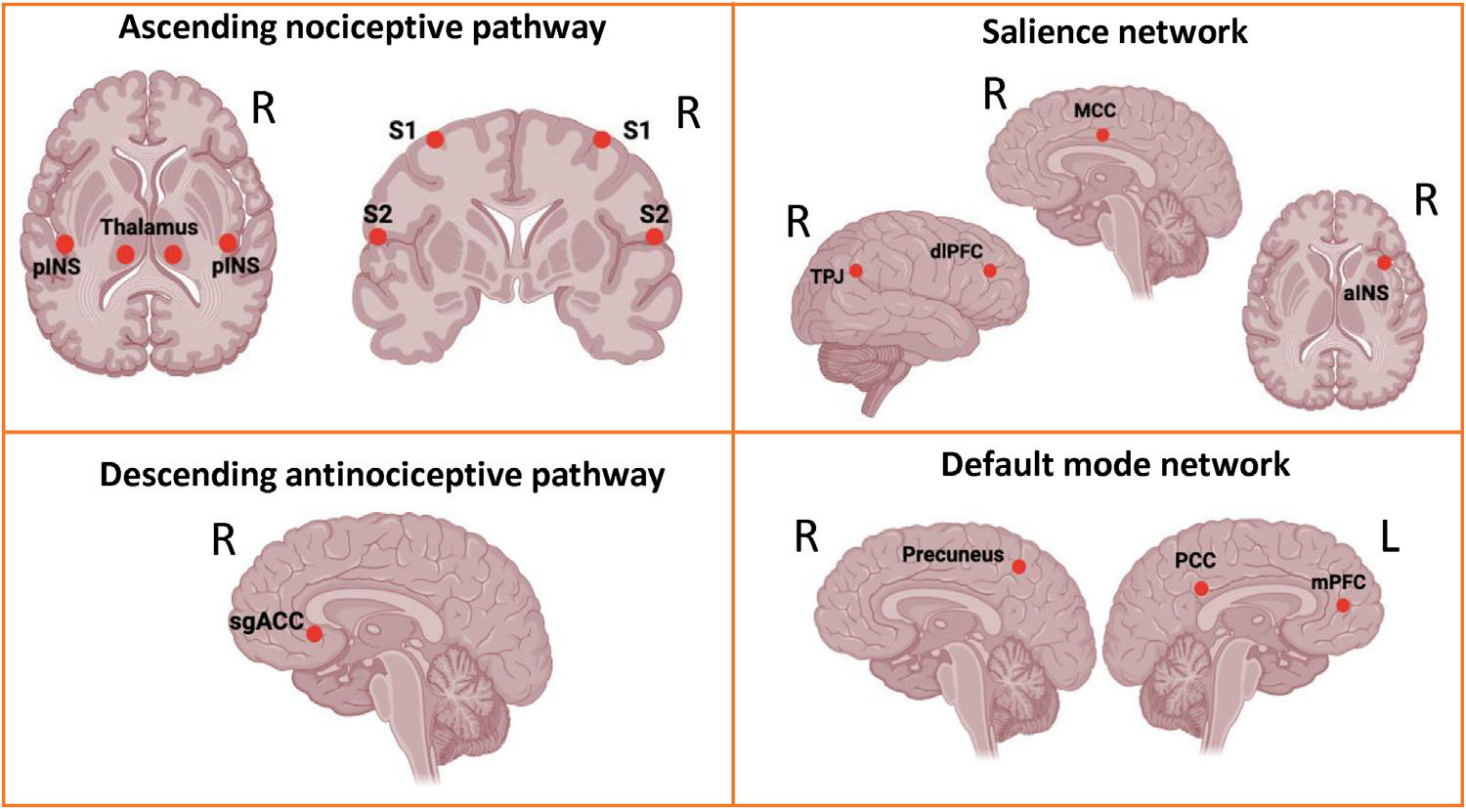
Regions of interest within the four networks of the dynamic pain connectome. aINS, anterior insula; dlPFC, dorsolateral prefrontal cortex; MCC, midcingulate cortex; mPFC, medial prefrontal cortex; PCC, posterior cingulate cortex; pINS, posterior insula; S1, primary somatosensory cortex; S2, secondary somatosensory cortex; sgACC, subgenual anterior cingulate cortex; TPJ, temporoparietal junction. Figure created with BioRender.com.

We selected regions of interest (ROIs) to represent key nodes within the four networks/pathways of the DPC based on previously defined coordinates (Fauchon et al. 2022b; Kim et al. 2019; Kisler et al. 2020; Kucyi and Davis 2015). These coordinates were visually validated on a Montreal Neurological Institute's 152‐brain standard template (MNI152). The following are the MNI coordinates (*x*, *y*, *z*) for the selected ROIs (see Figure 2): (1) ascending nociceptive pathways: left thalamus (−12, −18, 8), right thalamus (12, −18, 8), left primary somatosensory cortex (S1) (−34, −30, 54), right S1 (34, −28, 54), left secondary somatosensory cortex (S2) (−60, −30, 20), right S2 (60, −22, 18), left posterior insula (−34, −20, 18), right posterior insula (34, −20, 18); (2) descending antinociceptive pathway: subgenual anterior cingulate cortex (sgACC) (4, 26, −8); (3) SN: right temporoparietal junction (TPJ) (50, −32, 28), right anterior insula (34, 18, 4), mid‐cingulate cortex (MCC) (2, 12, 34), right dorsolateral prefrontal cortex (dlPFC) (34, 46, 22); (4) DMN: posterior cingulate cortex (PCC) (−2, −46, 28), medial prefrontal cortex (mPFC) (−2, 50, 2), precuneus (2, −61, 48).

### 2.5 MEG Preprocessing and Power Spectra Analysis

The preprocessing of the resting‐state MEG data followed established methodologies previously described in detail (Fauchon et al. 2022b; Kim et al. 2019; Kisler et al. 2020). Utilizing the FieldTrip toolbox (http://www.fieldtriptoolbox.org/), a MATLAB‐based software (MATLAB R2021a), the data underwent bandpass filtering within the range of 1–150 Hz, with a notch filter applied at 60 and 120 Hz. Independent component analysis (ICA) using the “runica” algorithm was applied to the continuous non‐segmented time‐series data to remove artefacts associated with eye blink, cardiac artefact, breathing, and muscle activity. To co‐register the MEG scan with the anatomic MRI for each individual, the fiducial points obtained prior to the MEG scan were located on each participant's anatomical MRI. Afterward, the single‐shell model was used for forward modelling.

The ROIs within the DPC were precisely defined, and a linearly constrained minimum variance (LCMV) beamformer was used to extract continuous time‐series data from these ROIs (Sekihara et al. 2001; van Veen et al. 1997). After completion of the beamforming process, the resting‐state time‐series for each individual was normalized to a *z*‐score to account for individual variability in the raw magnitude of relative power spectra and to allow group‐level inference. The theta band was defined as 4–8 Hz oscillations and the alpha band was defined as 8–13 Hz oscillations.

**FIGURE 3:**
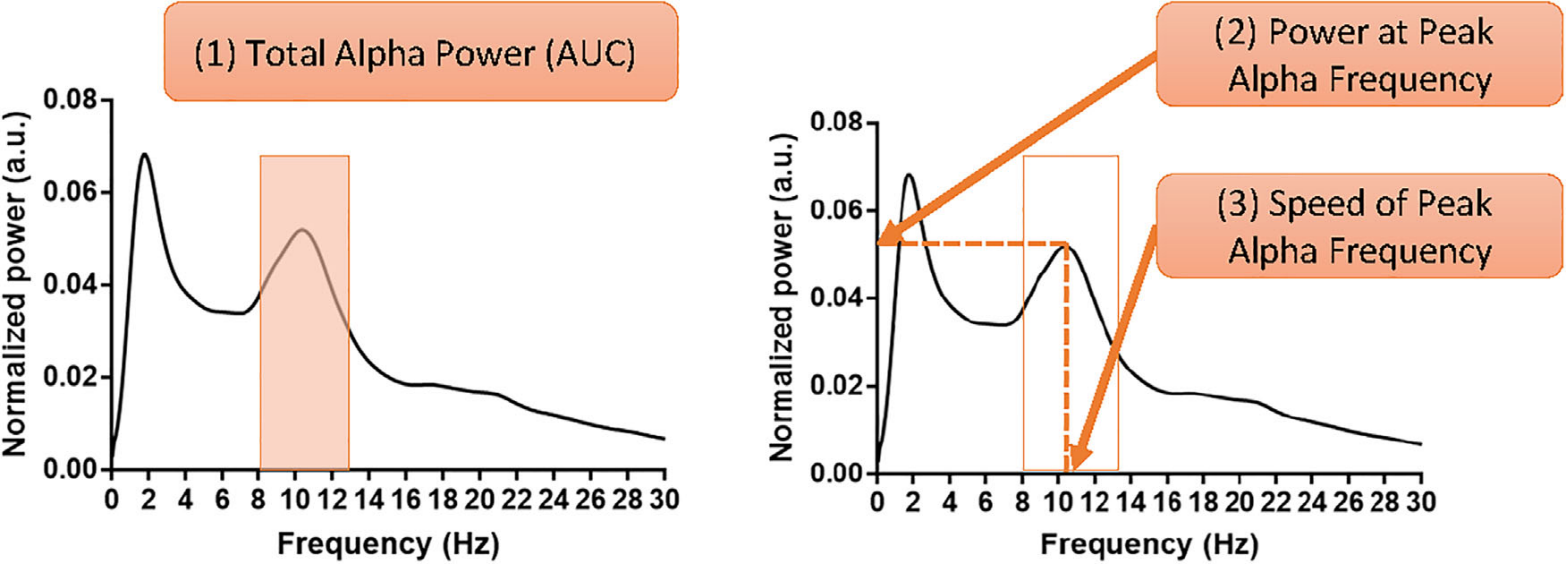
Metrics of attributes of alpha oscillations. Three metrics that were used to evaluate alpha oscillation: (1) total alpha power, (2) power at peak alpha frequency, and (3) speed of peak alpha frequency. AUC, area under the curve.

Power spectra analysis was performed for each ROI using the Welch's power density estimate (pwelch function in MATLAB) on the normalized resting‐state time‐series. The window length was set to 1000 samples with 50% overlap between consecutive windows. This configuration resulted in frequency intervals of 0.1 Hz. Three metrics were used for measuring alpha oscillations. These were total alpha power, PAF speed, and PAF power (Figure 3). PAF was assessed individually for each subject by identifying the maximum power restricted to the alpha band (8–13 Hz). The area under the curve (AUC) was calculated for the alpha (8–13 Hz) and theta (4–8 Hz) ranges using left‐point Riemann sum.

### 2.6 Statistical Testing

We used Mann–Whitney *U* tests to evaluate subgroups (A vs. P type/low vs. high IAP) to compare the group averages of PAF speed, power at PAF, total alpha/theta power at each ROI. *p* values were adjusted for multiple comparisons according to the number of ROIs using false discovery rate (FDR) correction with Benjamin–Hochberg method at FDR < 0.05 across ROIs. Cohen's *d* was used to calculate effect sizes for each Mann–Whitney *U* test. Also, Spearman's correlations were conducted for each ROI to examine the relationship between IAP score and PAF speed/power or ΔRT_mean_ and PAF speed/power.

## 3 Results

### 3.1 IAP Behavioral Results

**FIGURE 4:**
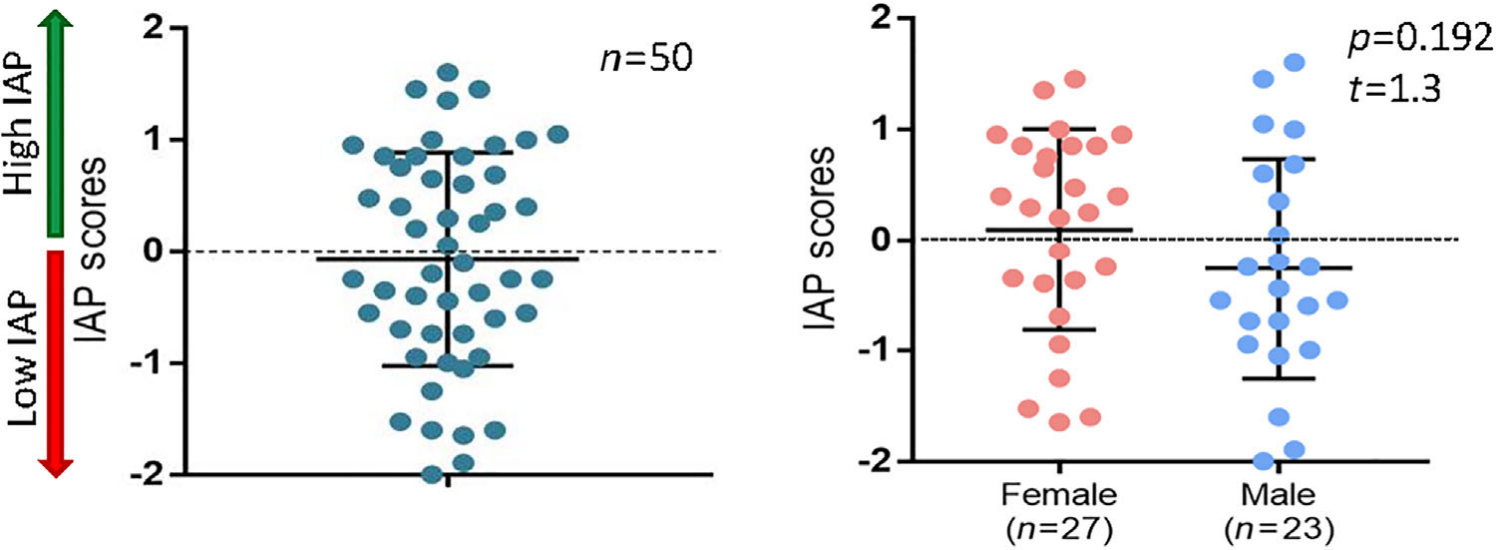
Distribution of individual intrinsic attention to pain (IAP) scores. IAP scores are shown for all 50 participants (left panel) and female and male subgroups (right panel).

The IAP scores of the participants in this study ranged from −2.0 to +1.6 (mean ± SD = −0.07 ± 0.95; *N* = 50) and were normally distributed. In addition, there were no statistically significant sex differences in IAP scores (mean_females_ ± SD = 0.09 ± 0.91; mean_males_ ± SD = −0.26 ± 0.99; *t* = 1.3, *p* = 0.192; *n*_female_ = 27, *n*_male_ = 23; Figure 4).

### 3.2 Association Between Alpha Oscillations and IAP

**FIGURE 5:**
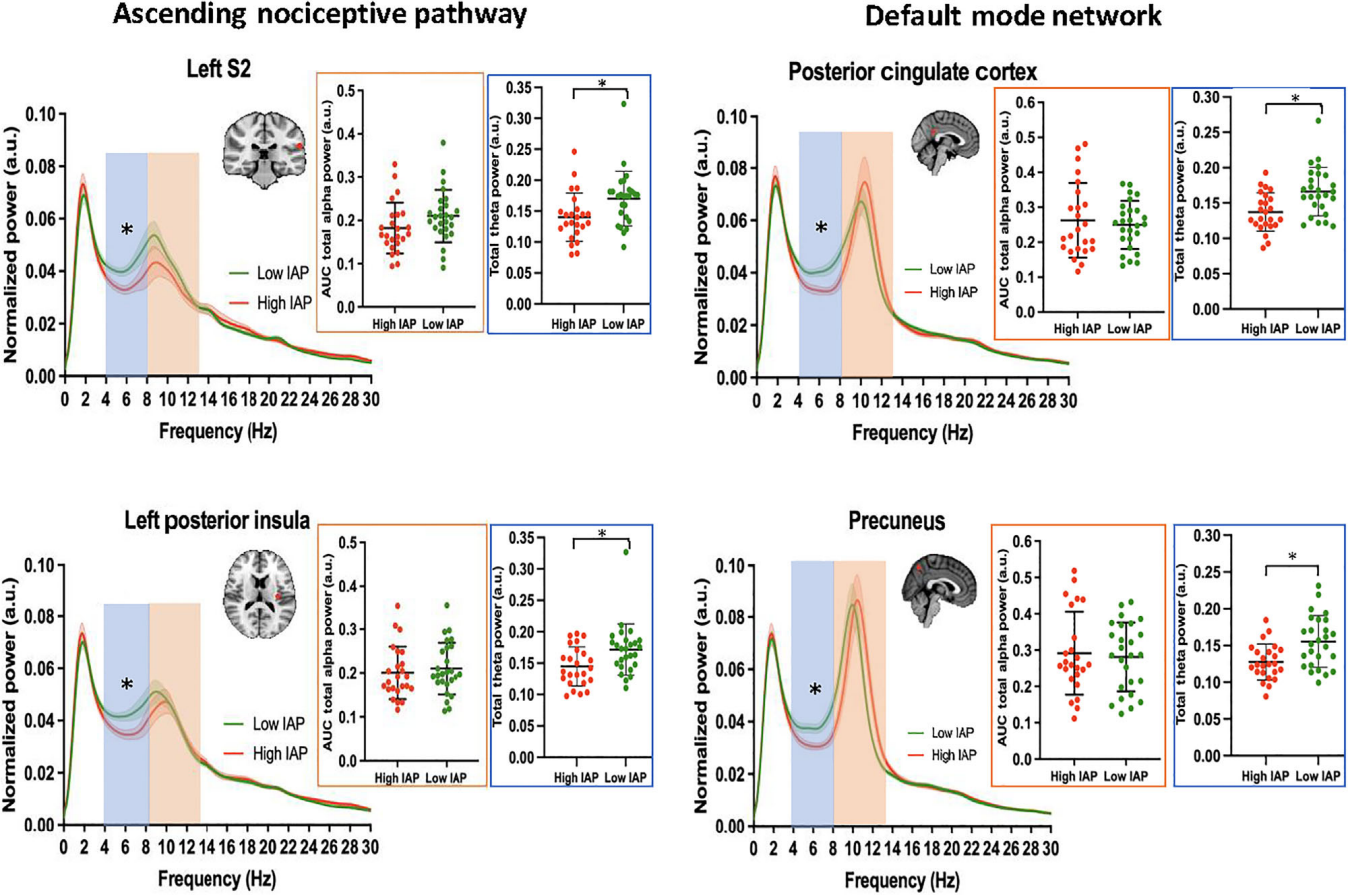
Power spectra comparisons between low (green) and high (red) IAP groups in selected nodes of ascending nociceptive pathway and default mode network. Mean ± SEM of normalized MEG power for each group and AUC comparisons for alpha and theta range are shown. Orange and blue shadings represent the AUC for alpha and theta oscillations, respectively. AUC, area under curve; IAP, intrinsic attention to pain. *p values significant after correcting for multiple comparison.

Three metrics of alpha activity were used: the total alpha power (AUC for 8–13 Hz) was not statistically different (*P*_FDR_ > 0.05) between the low‐IAP group (i.e., those with IAP score less than 0) and the high‐IAP group (i.e., those with IAP score more than 0) with small or negligible effect sizes (Figures 5, S1, and S2). Two approaches were used to investigate whether mind‐wandering away from pain is reflected by the speed or power of PAF within key nodes of DPC. The first approach involved group comparison analyses, comparing PAF between low‐ and high‐IAP groups. The second approach included correlation analyses, aiming to uncover potential relationships between individual IAP scores and PAF within each ROI in the DPC.

**FIGURE 6:**
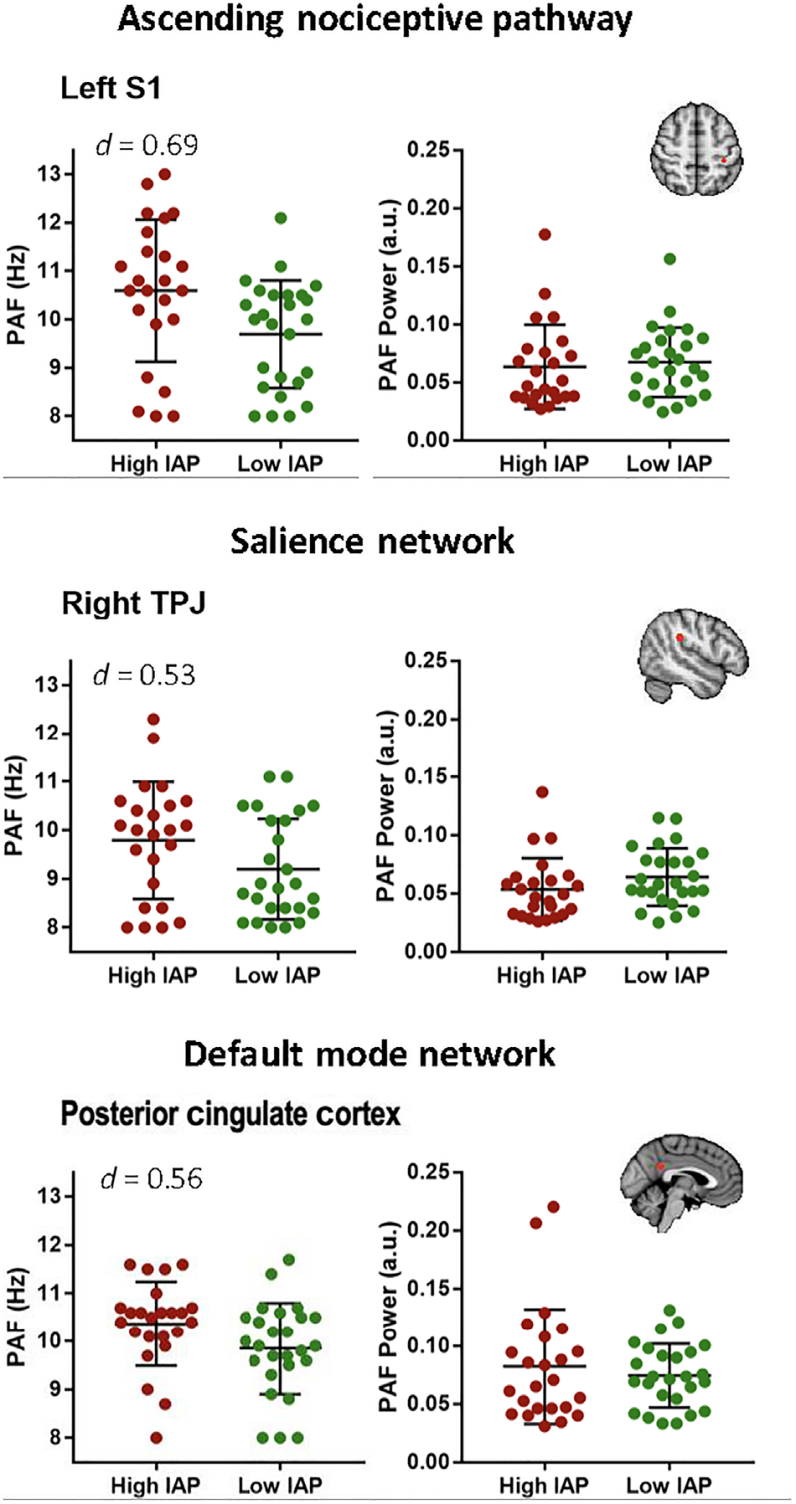
Group comparison of PAF speed and power between high (red) and low (green) IAP. Mean ± SD of PAF for each group are shown for left S1, right TPJ, and posterior cingulate cortex each belonging to ascending nociceptive pathway, salience network, and default mode network, respectively. IAP, intrinsic attention to pain; PAF, peak alpha frequency; S1, primary somatosensory cortex; TPJ, temporoparietal junction.

After correcting *p* values for multiple comparison, the PAF speed was not significantly different (*P*_FDR_ > 0.05) between the two groups in any of the ROIs. Some examples of these comparisons are shown in Figure 6 (results for all ROIs shown in Figure S3). However, there were notable medium effect sizes in the left S1 (*d* = 0.69) node of the ascending nociceptive pathway, the PCC (*d* = 0.56), and the precuneus (*d* = 0.55) nodes within the DMN which suggest that the PAF within the low‐IAP group was slower compared to the high‐IAP individuals. The correlation analyses showed no correlation between IAP score and PAF speed in any of the ROIs after correction for multiple comparisons (Figure S4). In addition, the PAF power was not significantly different (*P*_FDR_ > 0.05) between the two groups in any of the ROIs. Examples are depicted in Figure 6 (results for all ROIs shown in Figure S5). After correcting for multiple comparisons, there was no significant correlation between individual IAP scores and the power of PAF. For correlation graphs, see Figure S6.

### 3.3 Association Between Theta Oscillations and IAP

Brain–behavioral relationships were assessed and between‐group comparisons were made for the low‐ and high‐IAP groups. There were notable group differences (*P*_FDR_ < 0.05) in total theta power (AUC for 4–8 Hz) within nodes of ascending nociceptive pathway and DMN. Specifically, compared to those with high‐IAP, low‐IAP individuals had significantly greater total theta power with medium effect size in nodes of ascending nociceptive pathway: left thalamus (*P*_uncor_ = 0.0174, *P*_FDR_ = 0.0464, *d* = 0.71), right thalamus (*P*_uncor_ = 0.0164, *P*_FDR_ = 0.0464, *d* = 0.64), left S2 (*P*_uncor_ = 0.0117, *P*_FDR_ = 0.0464, *d* = 0.72), left posterior insula (*P*_uncor_ = 0.0139, *P*_FDR_ = 0.0464, *d* = 0.73), right posterior insula (*P*_uncor_ = 0.0216, *P*_FDR_ = 0.0494, *d* = 0.64) (Table 2). In addition, compared to those with high‐IAP, low‐IAP individuals had significantly greater total theta power with large effect size in nodes of DMN: PCC (*P*_uncor_ = 0.0029, *P*_FDR_ = 0.0368, *d* = 0.93) and precuneus (*P*_uncor_ = 0.0046, *P*_FDR_ = 0.0368, *d* = 0.91) (Table 2). Some examples of power spectra graphs and total theta power are shown in Figure 5. Comprehensive graphs for all nodes are included in Figures S1 and S2.

**TABLE 2:** Group differences of theta power between low‐ and high‐IAP individuals.

Network/pathway	Region of interest	Uncorrected *p* value	Corrected *p* value	Effect size
Ascending nociceptive pathway	Left thalamus	0.0174	0.0464^*^	0.71
	Right thalamus	0.0164	0.0464^*^	0.64
	Left S1	0.0326	0.0576	0.66
	Right S1	0.1605	0.1834	0.55
	Left S2	0.0117	0.0464^*^	0.72
	Right S2	0.0553	0.0737	0.55
	Left posterior insula	0.0139	0.0464^*^	0.73
	Right posterior insula	0.0216	0.0494^*^	0.64
Descending antinociceptive pathway	sgACC	0.0971	0.1195	0.54
Salience network	Right TPJ	0.0253	0.0506	0.69
	Right anterior insula	0.0360	0.0576	0.57
	Midcingulate cortex	0.0504	0.0733	0.63
	Right dlPFC	0.3698	0.3698	0.28
Default mode network	PCC	0.0029	0.0368^*^	0.93
	mPFC	0.2197	0.2343	0.34
	precuneus	0.0046	0.0368^*^	0.91

Abbreviations: dlPFC, dorsolateral prefrontal cortex; mPFC, medial prefrontal cortex; PCC, posterior cingulate cortex; S1, primary somatosensory cortex; S2, secondary somatosensory cortex; sgACC, subgenual anterior cingulate cortex; TPJ, temporoparietal junction.

* Significant *p* values after controlling for multiple comparisons.

### 3.4 A/P Behavioral Results

**FIGURE 7:**
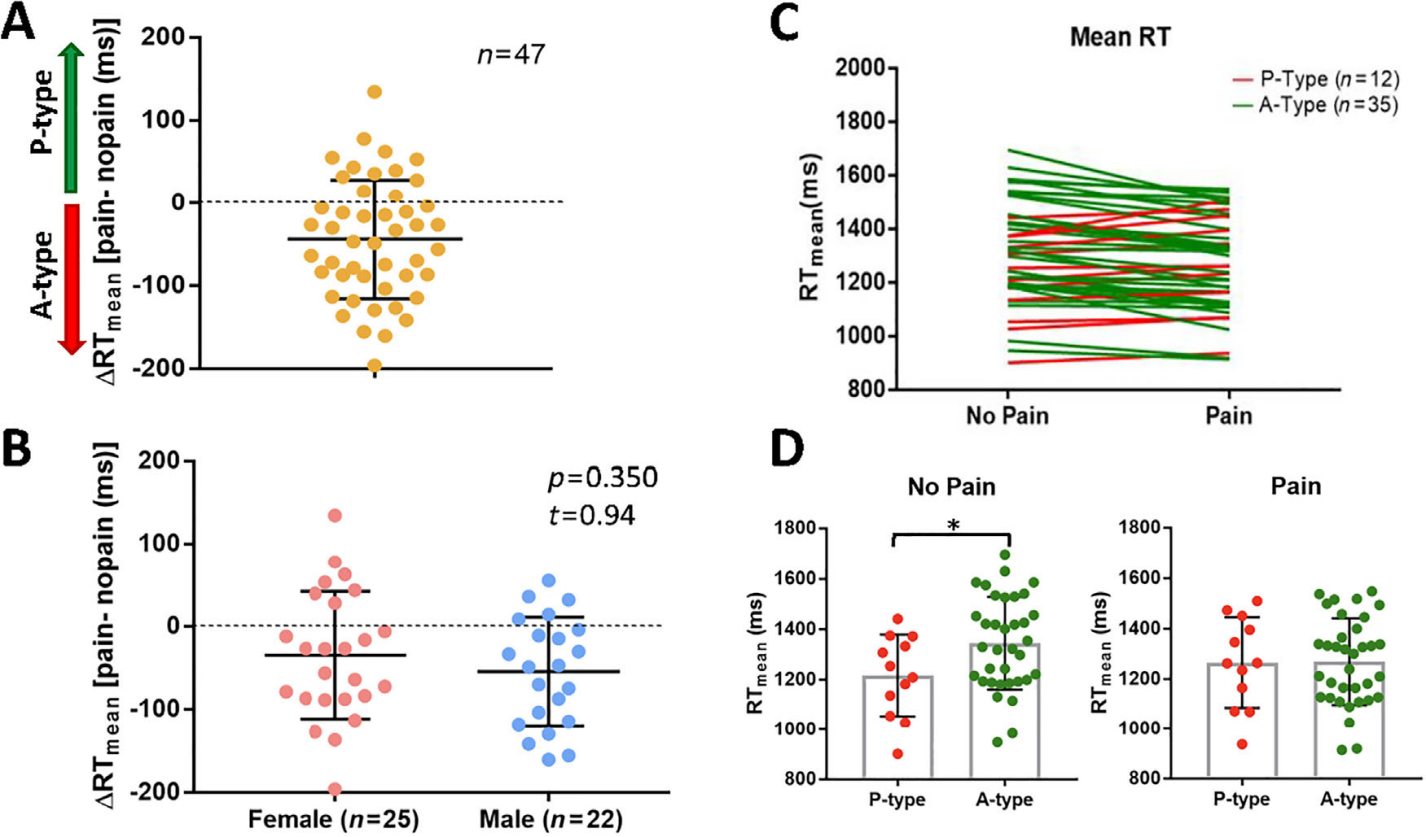
Reaction times (RTs). (A) The distribution of RT differences between the pain and no‐pain conditions for the entire participant cohort. (B) Sex‐segregated ΔRT_mean_. (C) Average reaction time in the no‐pain and pain block for each participant. Subjects classified as P‐ or A‐type whether their RT_mean_ increased or decreased during the pain block in comparison to the no‐pain block. (D) Mean reaction time shown for P‐ and A‐type individuals in no‐pain block on the left and pain block on the right. RT, reaction time; ΔRT_mean_, change in mean reaction time between pain and no‐pain condition (RT_mean pain_ − RT_mean no‐pain_). *p value group differences (*p* ≤ 0.05).

Task performance was quantified by RT and individuals were categorized as A or P type based on their ΔRT_mean_ (Figure 7A). There was no statistically significant sex difference in ΔRT_mean_ (mean_females_ ± SD = −34.77 ± 77.09; mean_females_ ± SD = −54.63 ± 65.43; *t* = 0.94, *p* = 0.350; *n*_female_ = 25, *n*_male_ = 22; Figure 7B). There were 35 A type and 12 P type individuals. P types were faster than A types during no‐pain blocks (mean_P‐type_ ± SD = 1216 ± 162 ms, *n* = 12; mean_A‐type_ ± SD = 1344 ± 184 ms, *n* = 35; *t* = 2.1, *p* = 0.037; Figure 7C,D). However, P and A types did not differ in mean RT during pain blocks (mean_P‐type_ ± SD = 1264 ± 180 ms, *n* = 12; mean_A‐type_ ± SD = 1269 ± 173 ms, *n* = 35; *t* = 0.07, *p* = 0.95; Figure 7C,D). Therefore, P types had pain‐induced slowing (mean_P‐type_ ± SD = 48 ± 33 ms, *n* = 12) whereas A types had pain‐induced increase in speed of task performance (mean_A‐type_ ± SD = −75 ± 50 ms, *n* = 35). In addition, across all the study participants, there was no statistically significant correlation between ΔRT_mean_ and IAP scores (rho = −0.004, *p* = 0.9775).

### 3.5 Association Between Alpha Oscillations and A/P

**FIGURE 8:**
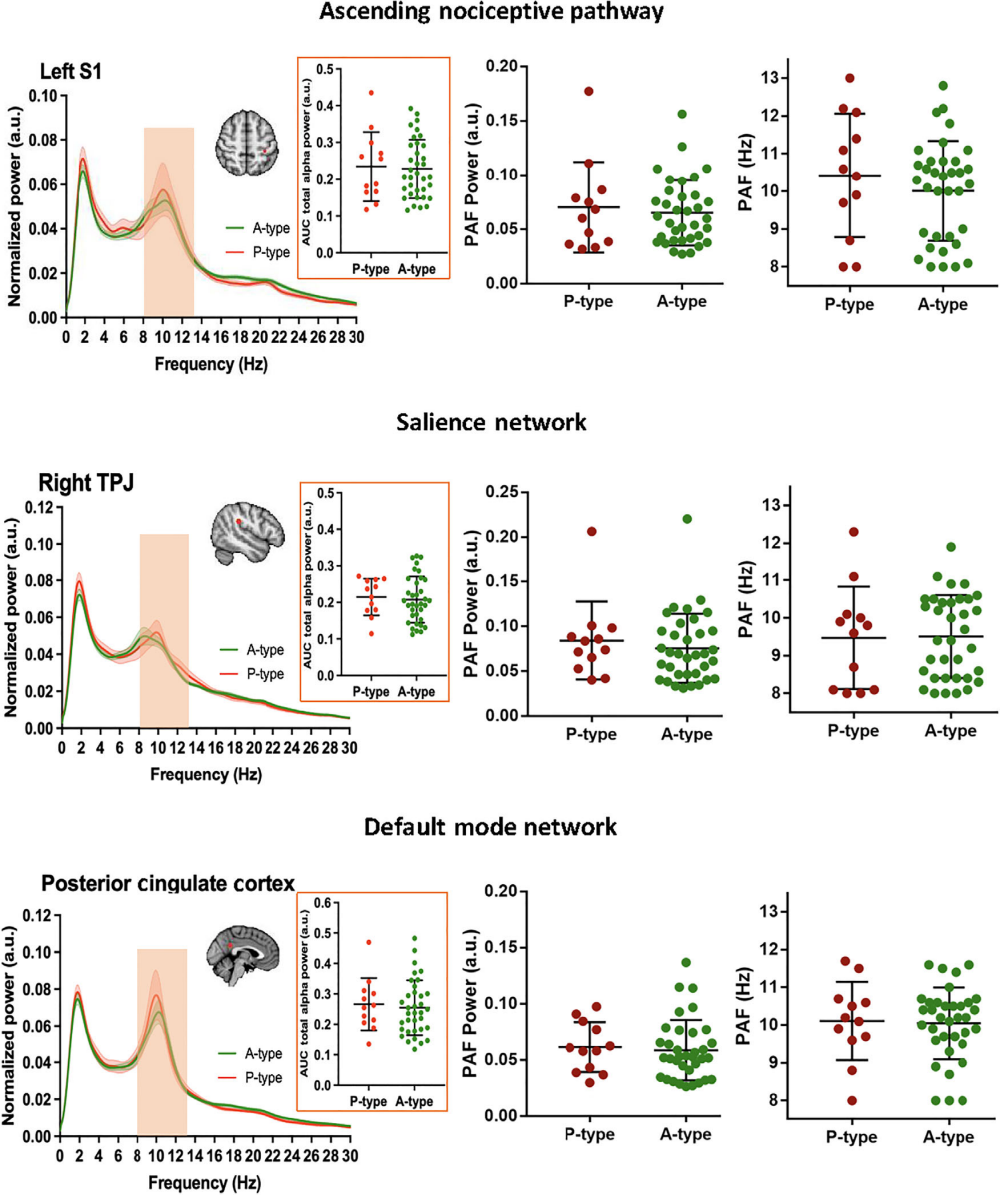
Power spectra comparisons between A‐ (green) and P‐ (red) type groups. Mean ± SEM of normalized MEG power for each group is shown for left S1, right TPJ, and posterior cingulate cortex belonging to ascending nociceptive pathway, salience network, and default mode network, respectively. PAF power and PAF speed are shown for the regions of interest. AUC, area under curve; S1, primary somatosensory cortex; TPJ, temporoparietal junction.

Total alpha power (AUC for 8–13 Hz) was not statistically different (*P*_FDR_ > 0.05) in any of the ROIs between A‐ and P‐type individuals (representative examples in Figures 8, S7, and S8). Two approaches were used to investigate whether pain impacting someone's cognitive performance during an attention‐demanding task is reflected by the speed or power of PAF within key nodes of DPC. The first approach involved group comparison analyses, comparing PAF between P‐ and A‐type individuals. The second approach included correlation analyses, aiming to uncover the potential relationship between ΔRT_mean_ and PAF within each ROI in the DPC.

PAF power/speed was not different (*P*_FDR_ > 0.05) between A‐ and P‐type individuals. Some examples are shown in Figure 8. For comprehensive graphs showing all ROIs, refer to Figures S9 and S10. In addition, the correlations between ΔRT_mean_ and PAF power/speed were not statistically significant (*P*_FDR_ > 0.05) at any of the ROIs (Figures S11 and S12).

### 3.6 Sex Differences

The IAP phenotype data were evaluated for sex differences. For the low‐IAP group (*n*_male_ = 15, *n*_female_ = 11) and the high‐IAP group (*n*_male_ = 8, *n*_female_ = 16), group comparison analyses were conducted to determine whether there was a sex difference in either PAF speed or PAF power within these groups (see Supporting Information Material for figures and tables pertaining to sex difference analyses). Within the low‐IAP groups, PAF speed did not show sex differences (*P*_FDR_ > 0.05) for any of the ROIs (Figure S13). However, in the low‐IAP group, PAF power was significantly higher (*P*_FDR_ < 0.05) in males than females across all ROIs, with large effect sizes (*d* ≥ 0.98) (Figure S14; Tables S1–S4). Within the high‐IAP group, in the right anterior insula, women had a faster PAF compared to men denoted by a large effect size (Cohen's *d* = 0.97) (Figure S15). For power at PAF, there were no sex differences (*P*_FDR_ > 0.05) in any of the ROIs, except for the right thalamus where there was a large effect size (Cohen's *d* = 1.37) denoting that males have higher power at PAF compared to females (Figure S16).

Regarding the A/P phenotype, for the A type individuals (*n*_male_ = 17, *n*_female_ = 18) and P type individuals (*n*_male_ = 5, *n*_female_ = 7), group comparison analyses were conducted to determine whether there was a sex difference in either PAF speed or PAF power within this group (Tables S5–S8). Within the A type individuals, the PAF speed did not show sex differences (*P*_FDR_ > 0.05) for any of the ROIs (Figure S17). For power at PAF, after correcting for multiple comparisons, PAF power was significantly different (*P*_FDR_ < 0.05) between sexes in the right thalamus (Figure S18). Also, large effect sizes denoted that males had higher PAF power in the right thalamus (*d* = 1.02), right S2 (*d* = 0.85), right posterior insula (*d* = 0.92), right TPJ (*d* = 0.97), right anterior insula (*d* = 0.74), and MCC (*d* = 0.85) compared to females. Within the P type individuals, there was no sex difference (*P*_FDR_ > 0.05) in PAF speed or power in any of the ROIs (Figures S19 and S20). However, large effect sizes showed males had higher PAF power in the left thalamus (*d* = 1.25), right thalamus (*d* = 1.27), right posterior insula (*d* = 1.74), right TPJ (*d* = 1.85), and precuneus (*d* = 01.66) compared to females.

## 4 Discussion

The IAP and A/P pain–attention phenotypes, characterized based on how individuals prioritize pain versus other attentional demands, have been associated with structural and functional MRI brain features (Cheng et al. 2017; Erpelding and Davis 2013; Kucyi, Salomons, and Davis 2013; Seminowicz, Mikulis, and Davis 2004). Building on this foundation, the present study used MEG to investigate the relationship between these phenotypes and the resting‐state alpha and theta oscillatory activity within the nodes of DPC. Although no significant differences were found in alpha oscillations between the subgroups within these phenotypes, distinct differences in the power of theta oscillations were observed among the two subgroups of the IAP phenotype.

The findings for total alpha power and PAF power showed no difference between low versus high‐IAP groups and A versus P types. The power at PAF represents the amplitude of neural activity at PAF, while total alpha power reflects the overall amount of alpha oscillatory activity across all frequencies within the 8–13 Hz alpha range. Given the proposed mechanism of alpha oscillations as “gating by inhibition,” higher alpha power in the nodes of a network might imply that there is an inherent baseline inhibitory state specific to that network (Jensen and Mazaheri 2010). In addition, it has been suggested that phasic inhibitory alpha oscillations allow reallocation of neural resources for information processing by silencing irrelevant parts of the cortex (van Diepen, Foxe, and Mazaheri 2019). Accordingly, although speculative, it could be that attentiveness to pain might be related to lower resting‐state alpha power in the ascending nociceptive pathway and SN. However, the findings of our paper indicate that the power of resting‐state inhibitory alpha activity in the DPC might not correlate with how an individual navigates or balances pain and attention needs. This is reasonable given that all participants in this study were healthy individuals, and it may be that aberrant or different power of resting‐state alpha activity occurs in a chronic pain state and not healthy individuals (Fauchon et al. 2022a, 2022b; Kim et al. 2019, 2020; Kisler et al. 2020). Therefore, the consistent alpha power in both ascending nociceptive and descending antinociceptive pathways within different groups of each pain–attention phenotype suggests that individuals within all these groups represent normal nociceptive‐related cortical network activity. This is in comparison to studies that have found higher alpha power in individuals with chronic pain conditions compared to healthy individuals (Fauchon et al. 2022a, 2022b; Kim et al. 2019; Kisler et al. 2020). In addition, a similar level of alpha activity within SN and DMN across IAP and A/P subgroups suggests that alpha activity in the DPC is responsive to various attentional demands, rather than being specifically attuned to pain. Again, this might represent a healthy condition of normal attentional circuitry as opposed to aberrant baseline resting‐state alpha rhythm that has been found in conditions such as attention‐deficit hyperactivity disorder (Deiber et al. 2020). It is important to recognize that there are no brain areas that are exclusively responsive to nociceptive stimuli or that are exclusively linked to pain perception (Iannetti et al. 2013; Wager et al. 2016). For instance, the SN has been shown to be non‐specific and activate in response to changes in different sensory stimuli (e.g., visual, auditory, tactile) (Downar et al. 2000, 2001, 2002). In addition, studies have shown that common brain regions are activated during pain perception and cognitive task performance and that regions supporting these two functions can be simultaneously active (Seminowicz and Davis 2007). Thus, overall baseline alpha activity might not differentiate whether an individual tends to prioritize one focus (e.g., task, mind‐wander) over another (e.g., pain).

Another metric for measuring alpha, aside from power, is the speed of PAF. This represents the most prominent speed of alpha activity and is thought to represent the rate at which sensory information is sampled (Cecere, Rees, and Romei 2015; Samaha and Postle 2015). The previous studies by Furman et al. (2018, 2020, 2019) presented a different perspective on the relationship between PAF and pain sensitivity: in a series of studies with independent cohorts, they showed that individuals with slower pain‐free resting‐state PAF tend to be more sensitive to prolonged pain elicited by two different pain models (e.g., phasic heat pain and capsaicin heat pain). Furman et al. (2018) attributed this finding to the different nature of painful stimuli (i.e., acute phasic pain vs. prolonged pain models). Moreover, they proposed that the finding of slower PAF predicting increased sensitivity to prolonged pain might by extension suggest that these individuals are more susceptible to the development of chronic pain (Furman et al. 2020). This is in line with the slowing of PAF in chronic pain patients in comparison to healthy individuals (Fauchon et al. 2022a, 2022b; Kim et al. 2019, 2020; Kisler et al. 2020; Lim et al. 2016). In light of these studies, the finding of this paper that low‐IAP individuals might have slower resting‐state PAF in left S1, PCC, and precuneus—highlighted by medium effect sizes—suggests that these individuals might have greater sensitivity to prolonged pain or be at risk of chronic pain. It may be counterintuitive that low‐IAP individuals could be more susceptible to develop chronic pain given that studies have identified hypervigilance to pain as a predictor of chronic pain development (Crombez et al. 2005; Lautenbacher et al. 2009). However, our understanding of how IAP relates to various measures of hypervigilance remains incomplete. In addition, it might be the case that for some individuals, a certain amount of attentiveness to pain‐related stimuli could act as a protective mechanism against the development of chronic pain. For example, in a study by Baum et al. (2011), it was observed that self‐reported hypervigilance to pain was significantly associated with pain sensitivity such that those with higher self‐reported hypervigilance to pain had lower experimental pain sensitivity (Baum et al. 2011). This finding might be consistent with the aforementioned rationale, emphasizing a potential protective role of attention to pain‐related stimuli against the development of chronic pain in a subset of individuals.

The findings of theta oscillations showed that in comparison to high‐IAP group, individuals with low‐IAP had significantly higher theta power within nodes of ascending nociceptive pathway (left S2, and bilateral posterior insula and thalamus) and of the DMN (PCC, precuneus nodes). It is not unexpected to see the involvement of theta power in pain–attention interaction given that theta oscillations are important in attentional processes (Helfrich et al. 2018; Kam et al. 2019; Keller, Payne, and Sekuler 2017). However, research on the role of theta in attention has yielded mixed results (Clayton et al. 2015). For instance, some studies report increased theta power during the performance of cognitive tasks, such as working memory and mental arithmetic (Hsieh and Ranganath 2014; Ishii et al. 2014). In contrast, other studies show an increase in theta amplitude during mind‐wandering episodes (Braboszcz and Delorme 2011; Rodriguez‐Larios and Alaerts 2021). However, all these studies converge in highlighting the involvement of theta oscillations in internally directed attention. Whether engaged in a cognitive task or mind‐wandering, both scenarios require a focus on internal mental processes rather than stimuli from the external environment. This is in line with studies that have shown increased theta activity being associated with internal attention (Cona et al. 2020; Magosso, Ricci, and Ursino 2021). In our study, we observed higher resting‐state theta power in low‐IAP individuals compared to high‐IAP individuals in the nodes of ascending nociceptive pathway and DMN. This increased theta power is in line with studies that have found increased theta during mind‐wandering episodes (Braboszcz and Delorme 2011; Kam et al. 2022; Rodriguez‐Larios and Alaerts 2021). In addition, Cona et al. (2020) discovered an association between increased theta power in DMN nodes (i.e., precuneus and PCC) and internally oriented attention. On the basis of these findings, it is plausible that individuals with low‐IAP have a predisposition for internally oriented attention (such as mind‐wandering) that is related to the higher resting‐state theta oscillation observed in the DMN nodes. Moreover, given the findings of increased theta power in individuals with chronic pain compared to healthy controls, it is interesting to consider that a low‐IAP classification might indicate an internally oriented attention style and consequently a susceptibility to develop chronic pain (Fallon et al. 2018; Sarnthein et al. 2006; Stern, Jeanmonod, and Sarnthein 2006; Walton, Dubois, and Llinas 2010).

In the sex difference analyses, within the low‐IAP group, men exhibited significantly higher PAF power at all ROIs within the nodes of DPC compared to women. However, there were no discernable sex differences within the high‐IAP group. Previously, Fauchon et al. (2022b) found that healthy males had higher PAF power compared to females. One plausible explanation for this effect not being observed in high‐IAP may be related to the observation that within the group of men with low‐IAP, there was less variation in PAF power compared to men in the high‐IAP group. This is likely due to the small sample size and might obscure sex differences in PAF power in the high‐IAP group. Therefore, interpretations of sex differences should be made with caution.

There are several limitations to bear in mind while interpreting this study. First, for the IAP analyses, participants' reports of “something else” were not further categorized into distinct types such as external sensory distractors, task‐related interferences, or mind‐wandering. This is important because in the study by Kucyi, Salomons, and Davis (2013), they observed that when subjects attended to external sensory distractors, they were unlikely to engage DMN, and when they reported mind‐wandering, they were more likely to engage DMN. Therefore, it might be that individuals within the low‐IAP group might not all have the tendency to “mind wander” and that they might be focusing on different distractors. This might be related to their resting‐state alpha oscillations, but we were not able to capture these potential differences because we grouped all of these individuals together. Second, source reconstruction for deep subcortical sources might be susceptible to co‐registration error and noise. Nevertheless, to improve confidence in inverse modeling, we implemented robust source reconstruction and artifact removal methods. Third, the recorded brain signals contain both oscillatory and non‐oscillatory (1/f) components. These components serve distinct functional roles and are likely generated by different neural mechanisms (Ouyang et al. 2020). This highlights the significance of decomposing these components for a more thorough analysis. However, we used the conventional power spectrum analysis that does not dissociate the oscillatory and non‐oscillatory components of the signal. Future studies should consider implementing spectral parameterization techniques, such as those proposed by Donoghue et al. (2020) to disentangle these two components and provide a more nuanced understanding of the role of alpha oscillations in the pain–attention interaction.

The focus of this paper is to evaluate the power of alpha and theta oscillations, specifically to capture the overall magnitude of neural activity within these frequency bands. To deepen our understanding of this relationship, future studies could use MEG data to assess functional coupling to determine the interdependence between brain regions. For example, cross‐frequency coupling (CFC) could be examined using the phase–amplitude coupling metric to explore the interactions between neural oscillations at different frequency bands. CFC can be analyzed both inter‐regionally (between different brain regions) and intra‐regionally (within a single brain region). Moreover, previous research has identified associations between PAF and pain sensitivity, as well as relationships between pain sensitivity and self‐reported hypervigilance (Baum et al. 2011; Furman et al. 2018, 2019, 2020). Ultimately, it would be interesting for future studies to explore how IAP scores relate to self‐reported hypervigilance and sensitivity to both acute versus prolonged pain models.

## 5 Conclusion

This study found that theta oscillation power in nodes of ascending nociceptive pathway and DMN is higher in individuals who tend to mind‐wander away from pain than in people who intrinsically attend to pain. In addition, there are no widespread or prominent brain–behavior relationships between alpha oscillations and the degree to which an individual pays attention to pain or how pain impacts an individual's cognitive performance during an attention‐demanding task. However, medium effect sizes showed PAF in the left S1, PCC, and precuneus being slower in low‐IAP individuals than in high‐IAP individuals. Therefore, alpha and theta oscillation within DPC may underlie aspects of pain–attention interactions.

## Author Contributions

**Nikou Kelardashti**: conceptualization, data curation, formal analysis, methodology, software, visualization, writing–original draft, writing–review and editing. **Benjamin T. Dunkley**: conceptualization, methodology, supervision, writing–review and editing. **Rima El‐Sayed**: investigation, writing–review and editing. **Vaidhehi Veena Sanmugananthan**: investigation, writing–review and editing. **Junseok Andrew Kim**: investigation, writing–review and editing. **Natalie Rae Osborne**: investigation, writing–review and editing. **Joshua C. Cheng**: investigation, writing–review and editing. **Anton Rogachov**: investigation, writing–review and editing. **Rachael L. Bosma**: investigation, writing–review and editing. **Ariana E. Besik**: investigation, writing–review and editing. **Karen Deborah Davis**: conceptualization, funding acquisition, project administration, resources, supervision, writing–original draft, writing–review and editing.

## Conflicts of Interest

The authors declare no conflicts of interest.

### Peer Review

The peer review history for this article is available at https://publons.com/publon/10.1002/brb3.70190.

## Supporting information



Supplementary Figures

Supplementary Tables

## Data Availability

The data that support the findings of this study are available on request from the corresponding author. The data are not publicly available due to privacy or ethical restrictions.
