# Supplementary material for: Alpha and Theta Oscillations Associated With Behavioral Phenotypes of Pain–Attention Interaction
Source: Brain Behav. 2025 Jan 19;15(1):e70190. doi: 10.1002/brb3.70190 (PMC11743985; doi:10.1002/brb3.70190)
Supplement: Supplementary file 1 — Supplementary Figures [file BRB3-15-e70190-s002.pdf]

Supplementary Figures

Alpha and Theta Oscillations associated with Behavioral Phenotypes of Pain-Attention Interaction

Nikou Kelardashti, Benjamin T. Dunkley, Rima El-Sayed, Vaidhehi Veena Sanmugananthan, Junseok Andrew Kim, Natalie Rae Osborne, Joshua C. Cheng, Anton Rogachov, Rachael L. Bosma, Ariana E. Besik, and Karen Deborah Davis

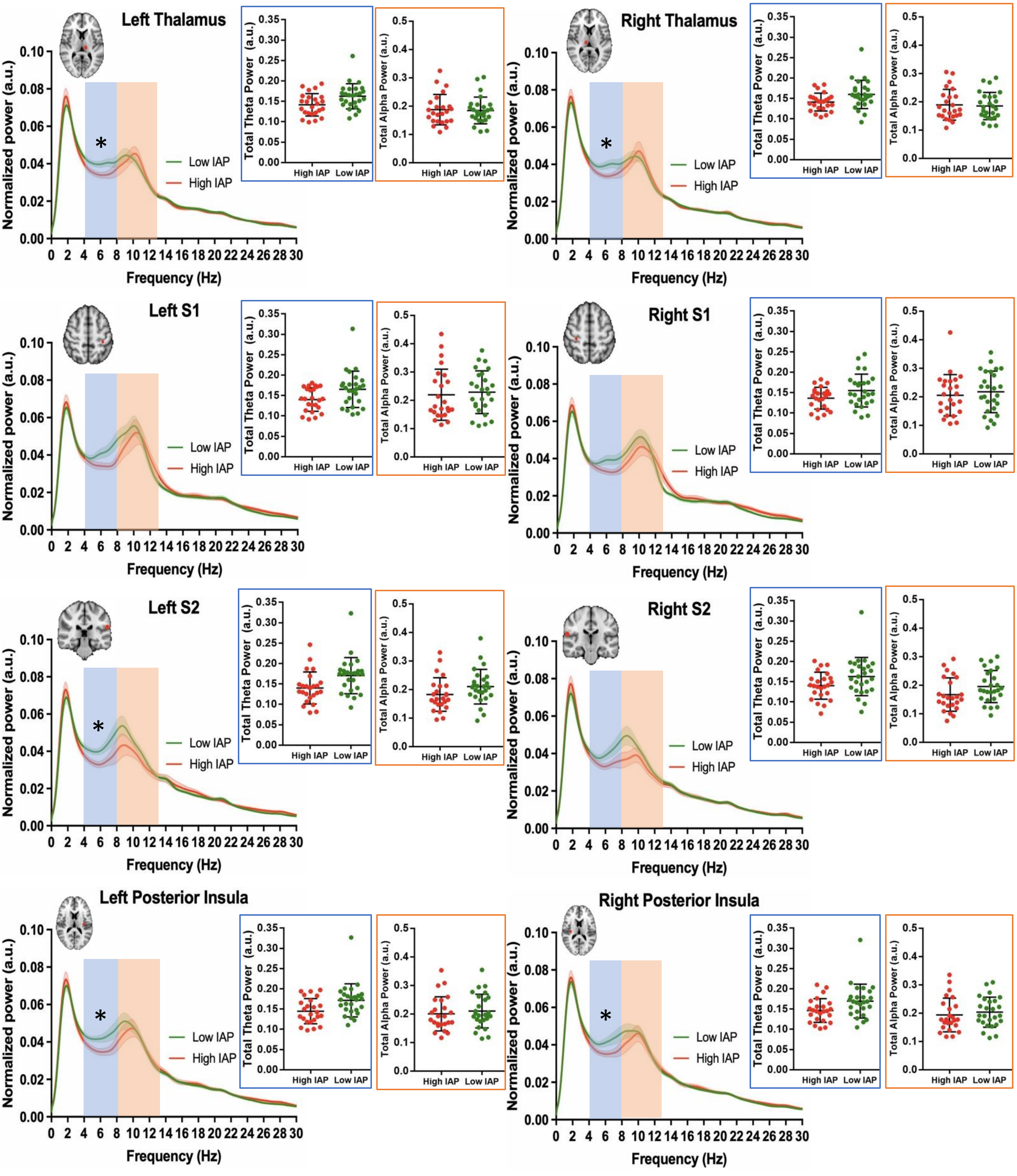

Supplementary Figure 1. Power spectra comparisons between low (green) and high (red) IAP groups in nodes of the ascending nociceptive pathway. The mean +/-SEM of normalized MEG power for each group and AUC comparisons for alpha and theta range are shown for key regions of interest within the ascending nociceptive pathway. Orange and blue shadings represent the AUC for alpha and theta oscillations respectively. *P-values significant after correcting for multiple comparison. IAP, intrinsic attention to pain; AUC, area under curve; S1, primary somatosensory cortex; S2, secondary somatosensory cortex.

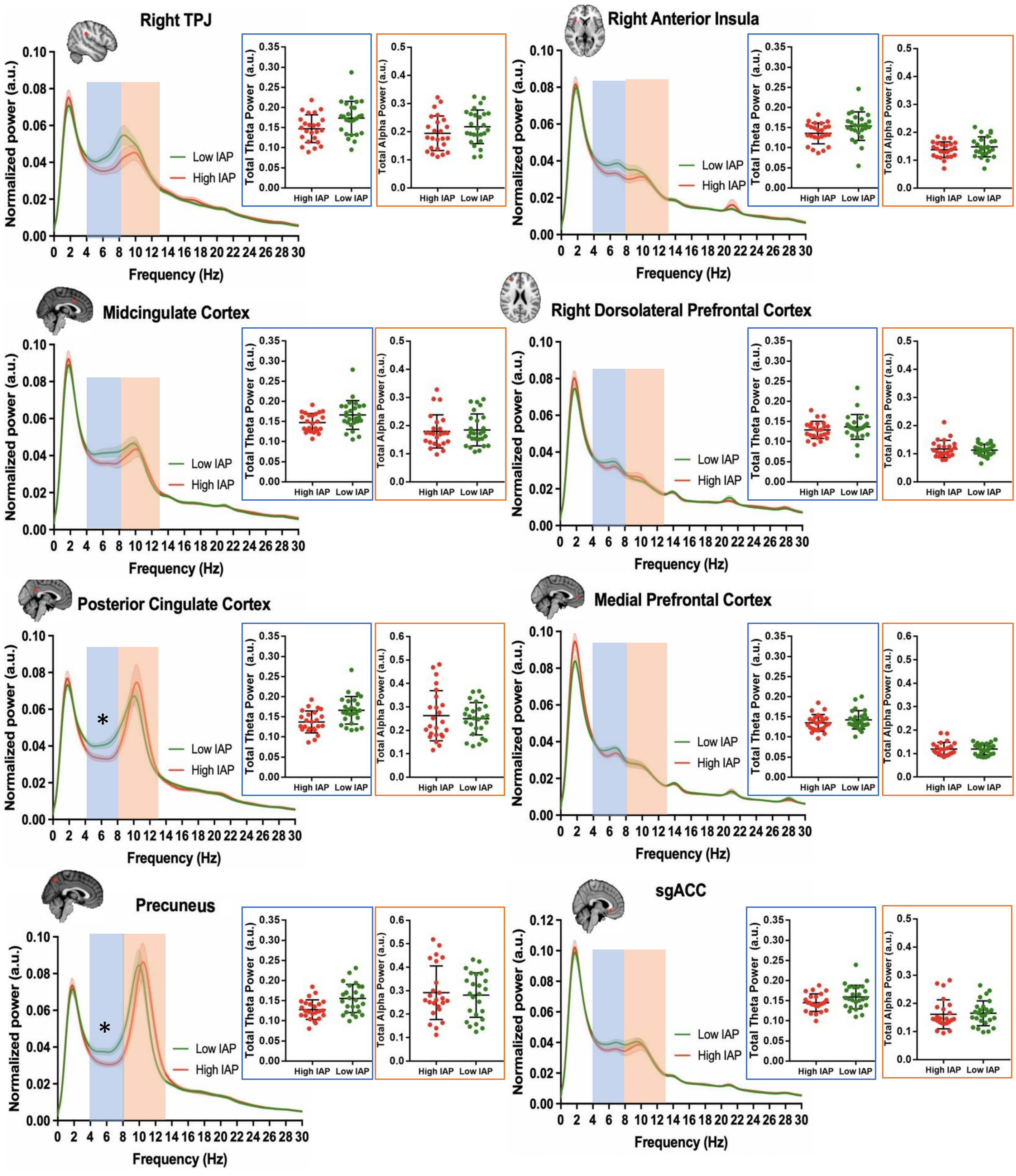

Supplementary Figure 2. Power spectra comparisons between low (green) and high (red) IAP groups in the nodes of salience network, default mode network, and descending antinociceptive pathway. The mean +/-SEM of normalized MEG power for each group and AUC comparisons for alpha and theta range are shown for key regions within the networks. Orange and blue bars represent the AUC for alpha and theta oscillations respectively. *P-values significant after correcting for multiple comparison. IAP, intrinsic attention to pain; AUC, area under curve; TPJ, temporoparietal junction; sgACC, subgenual anterior cingulate cortex.

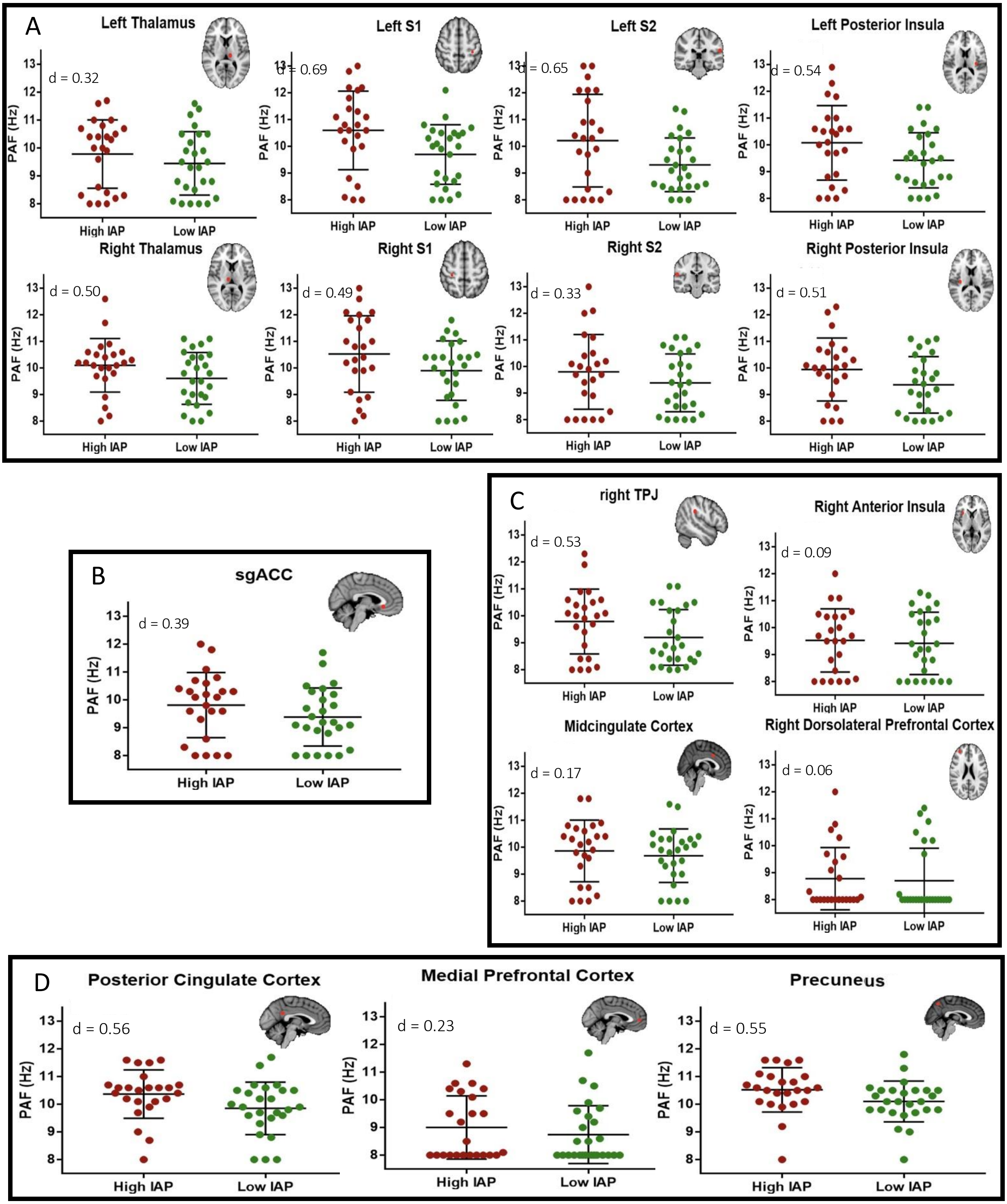

Supplementary Figure 3. Group comparison of PAF speed between high (red) and low (green) IAP. The mean +/- SD of PAF for each group are shown for key regions of interest within the A) ascending nociceptive pathway, B) descending antinociceptive pathway, C) salience network, and D) default mode network. IAP, intrinsic attention to pain; PAF, peak alpha frequency; S1, primary somatosensory cortex; S2, secondary somatosensory cortex; sgACC, subgenual anterior cingulate cortex; TPJ, temporoparietal junction.

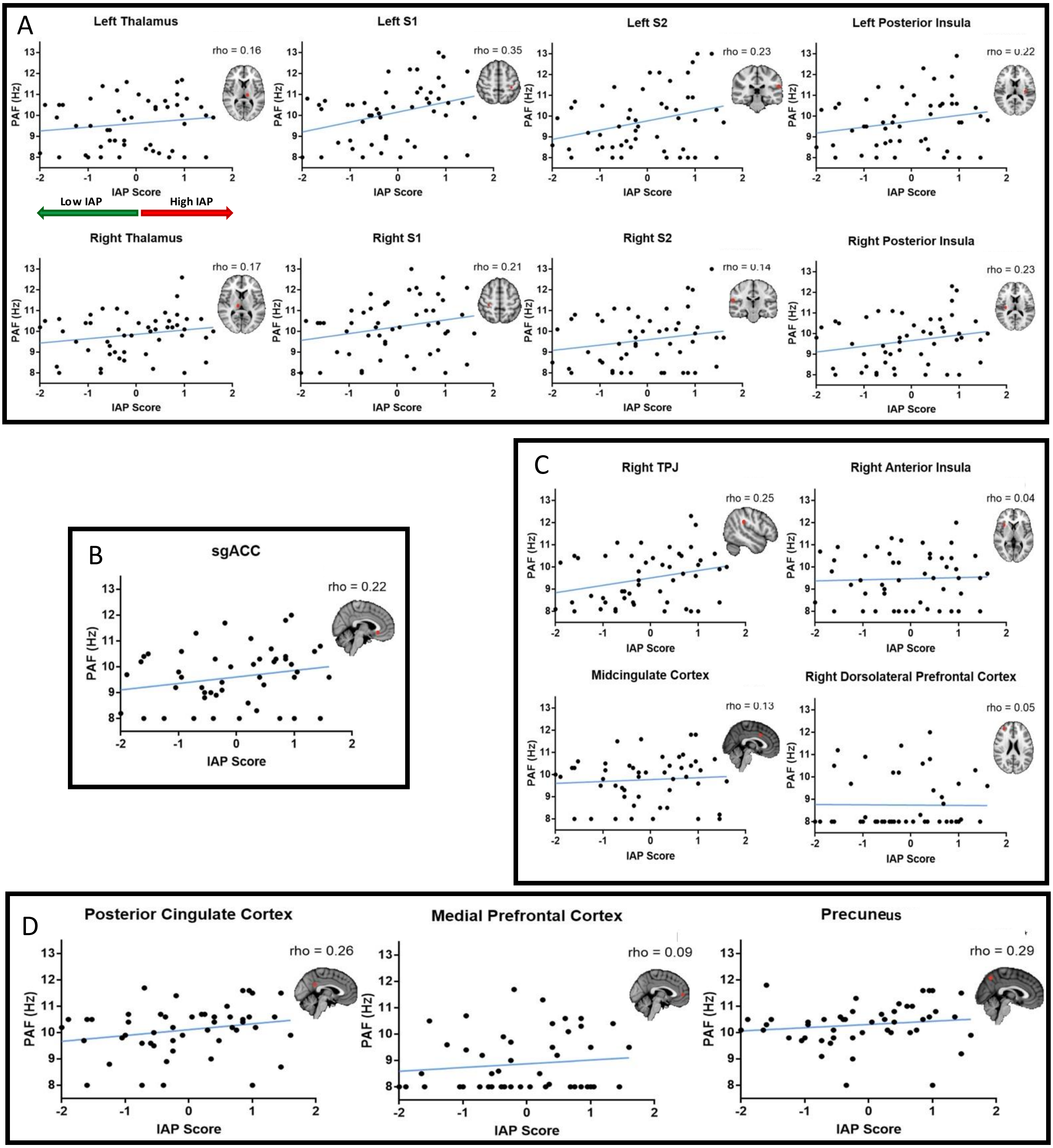

Supplementary Figure 4. Relationship between IAP scores and PAF speed. Panels A, B, C, D include ROIs within the ascending nociceptive pathway, descending antinociceptive pathway, salience network, and default mode network, respectively. IAP, intrinsic attention to pain; PAF, peak alpha frequency; S1, primary somatosensory cortex; S2, secondary somatosensory cortex; sgACC, subgenual anterior cingulate cortex; TPJ, temporoparietal junction.

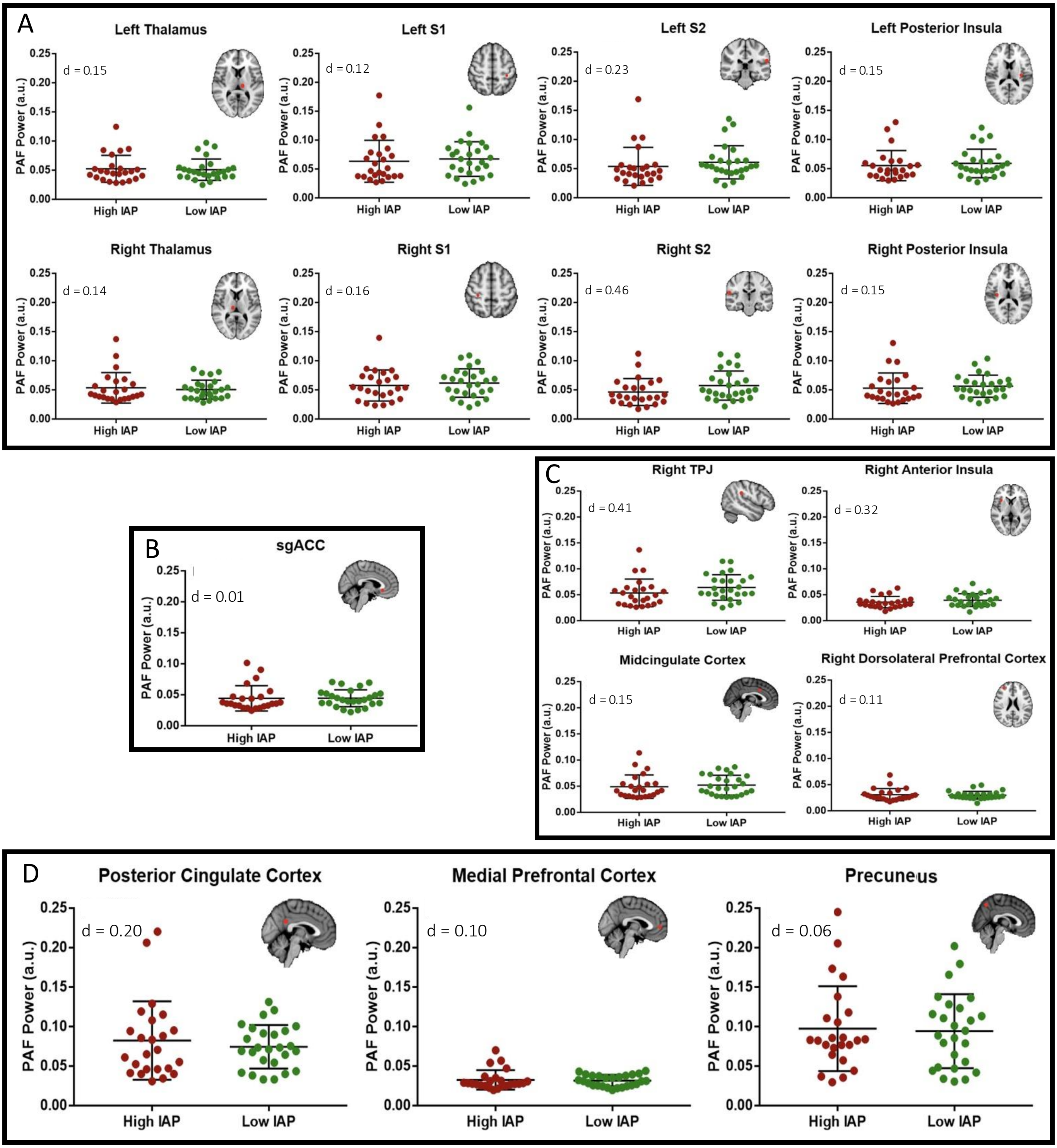

Supplementary Figure 5. Power spectra comparisons between low (green) and high (red) IAP groups in the nodes of salience network, default mode network, and descending antinociceptive pathway. The mean +/-SEM of normalized MEG power for each group and AUC comparisons for alpha and theta range are shown for key regions within the networks. Orange and blue bars represent the AUC for alpha and theta oscillations respectively. *P-values significant after correcting for multiple comparison. IAP, intrinsic attention to pain; AUC, area under curve; TPJ, temporoparietal junction; sgACC, subgenual anterior cingulate cortex

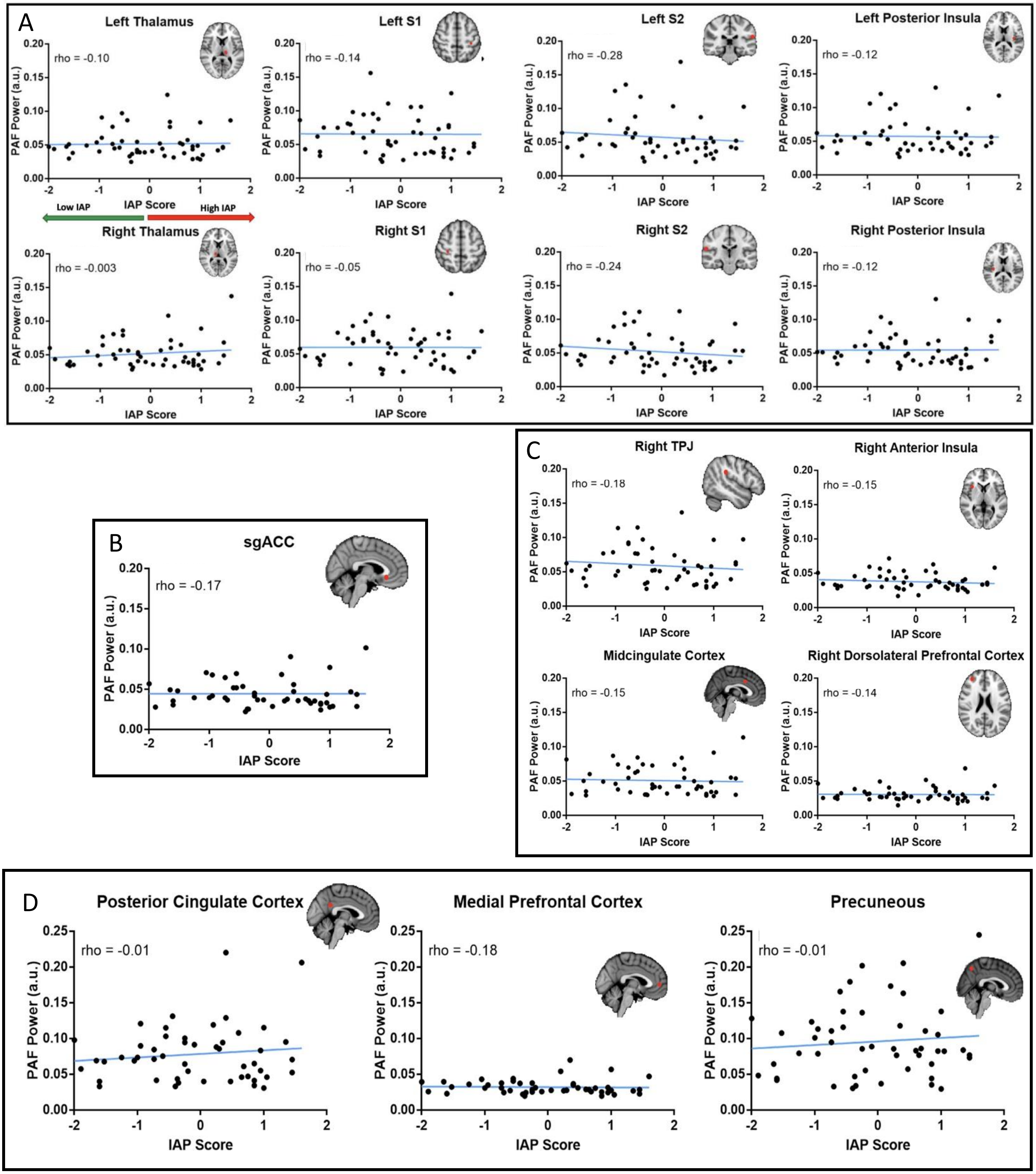

Supplementary Figure 6. Relationship between IAP scores and PAF power. Panels A, B, C, D include ROIs within the ascending nociceptive pathway, descending antinociceptive pathway, salience network, and default mode network, respectively. IAP, intrinsic attention to pain; PAF, peak alpha frequency; S1, primary somatosensory cortex; S2, secondary somatosensory cortex; sgACC, subgenual anterior cingulate cortex; TPJ, temporoparietal junction.

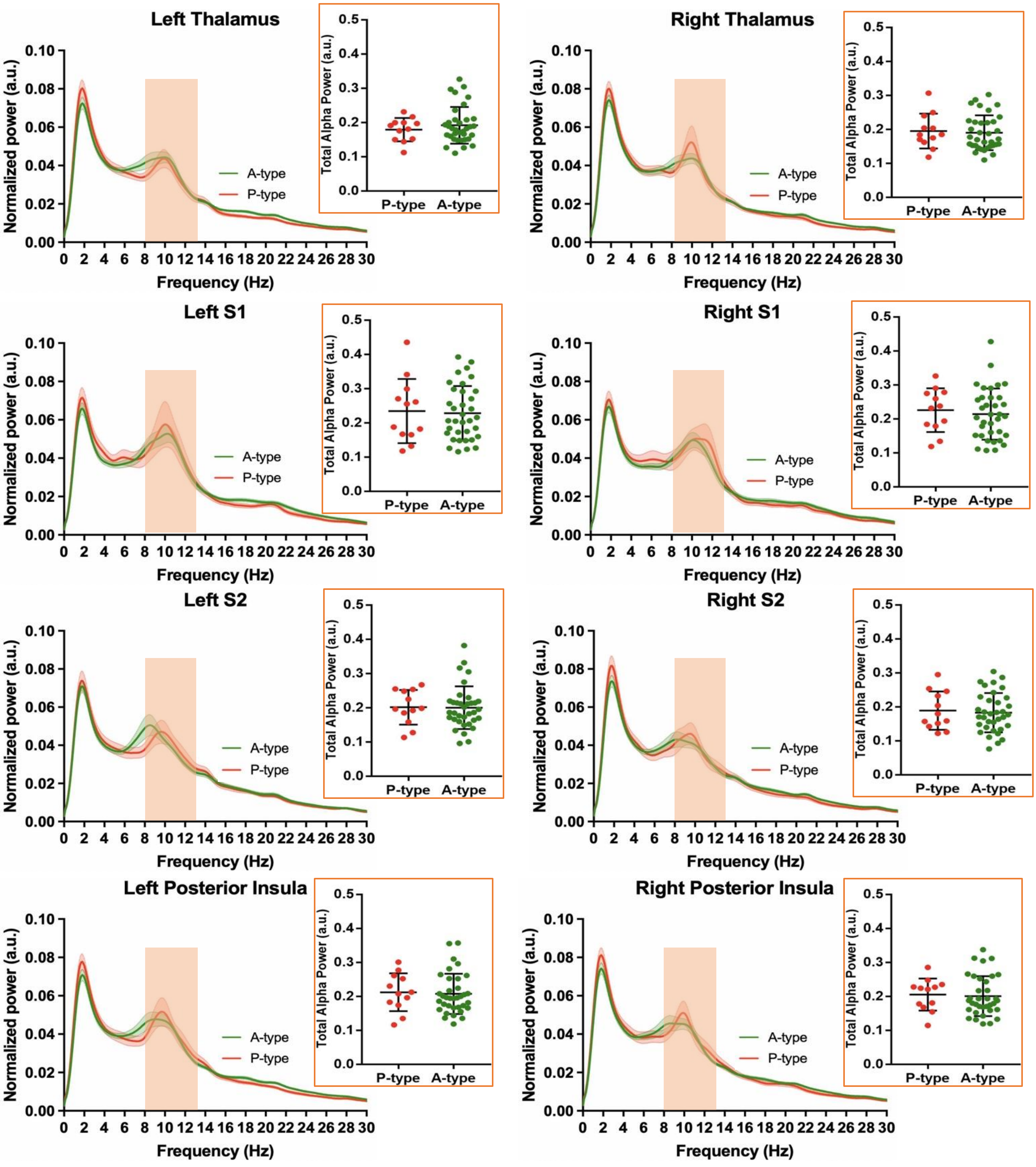

Supplementary Figure 7. Power spectra comparisons between A- (green) and P- (red) type groups in ascending nociceptive pathway. The mean +/-SEM of normalized MEG power for each group are shown for key regions of interest within the ascending nociceptive pathway. Reported P-values are uncorrected for multiple comparisons. S1, primary somatosensory cortex; S2, secondary somatosensory cortex; AUC, area under curve.

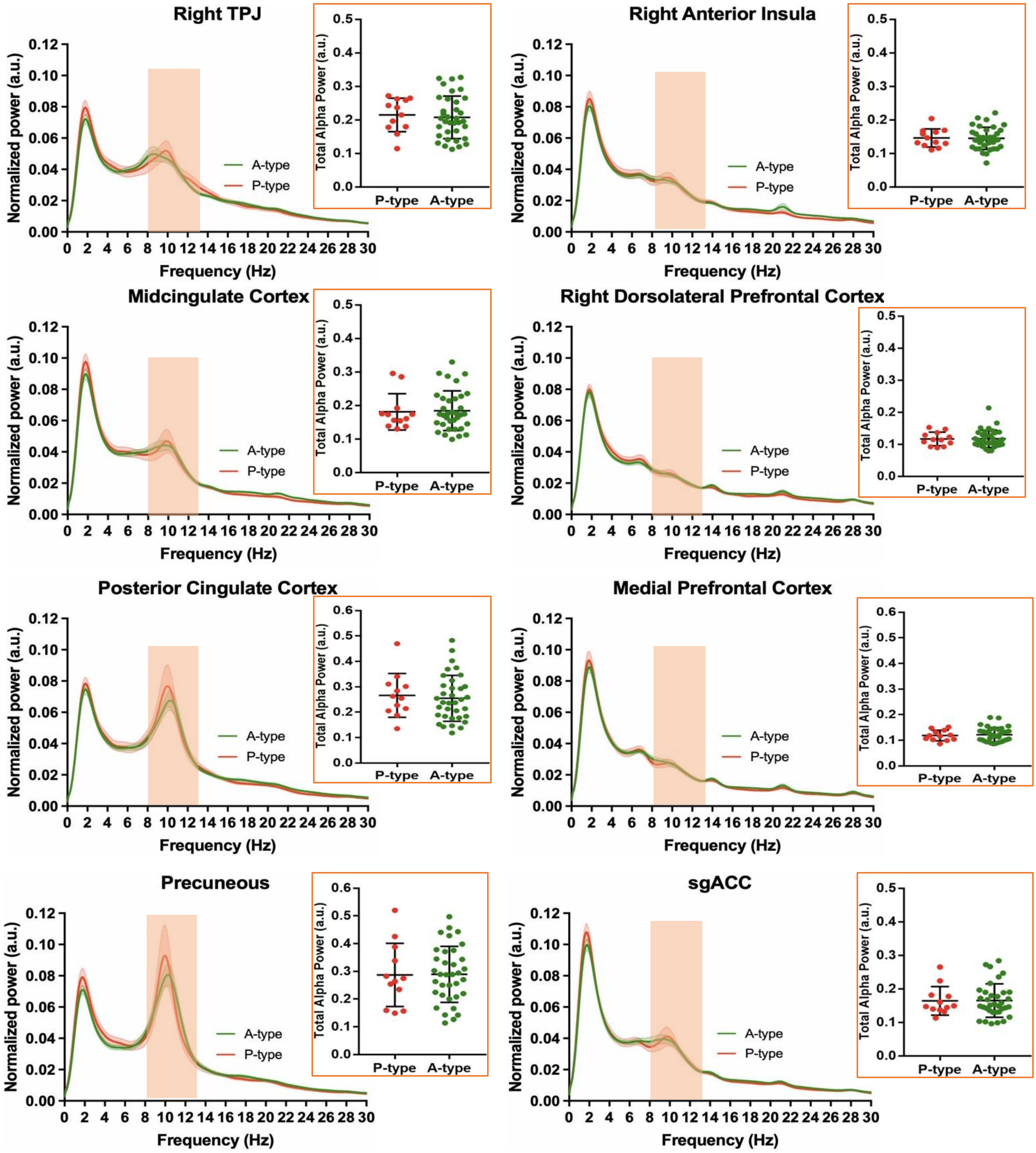

Supplementary Figure 8. Power spectra comparisons between A- (green) and P- (red) type groups in the nodes of salience network, default mode network, and descending antinociceptive pathway. The mean +/-SEM of normalized MEG power for each group are shown for key regions within the networks. Reported P-values are uncorrected for multiple comparisons. AUC, area under curve; TPJ, temporoparietal junction; sgACC, subgenual anterior cingulate cortex.

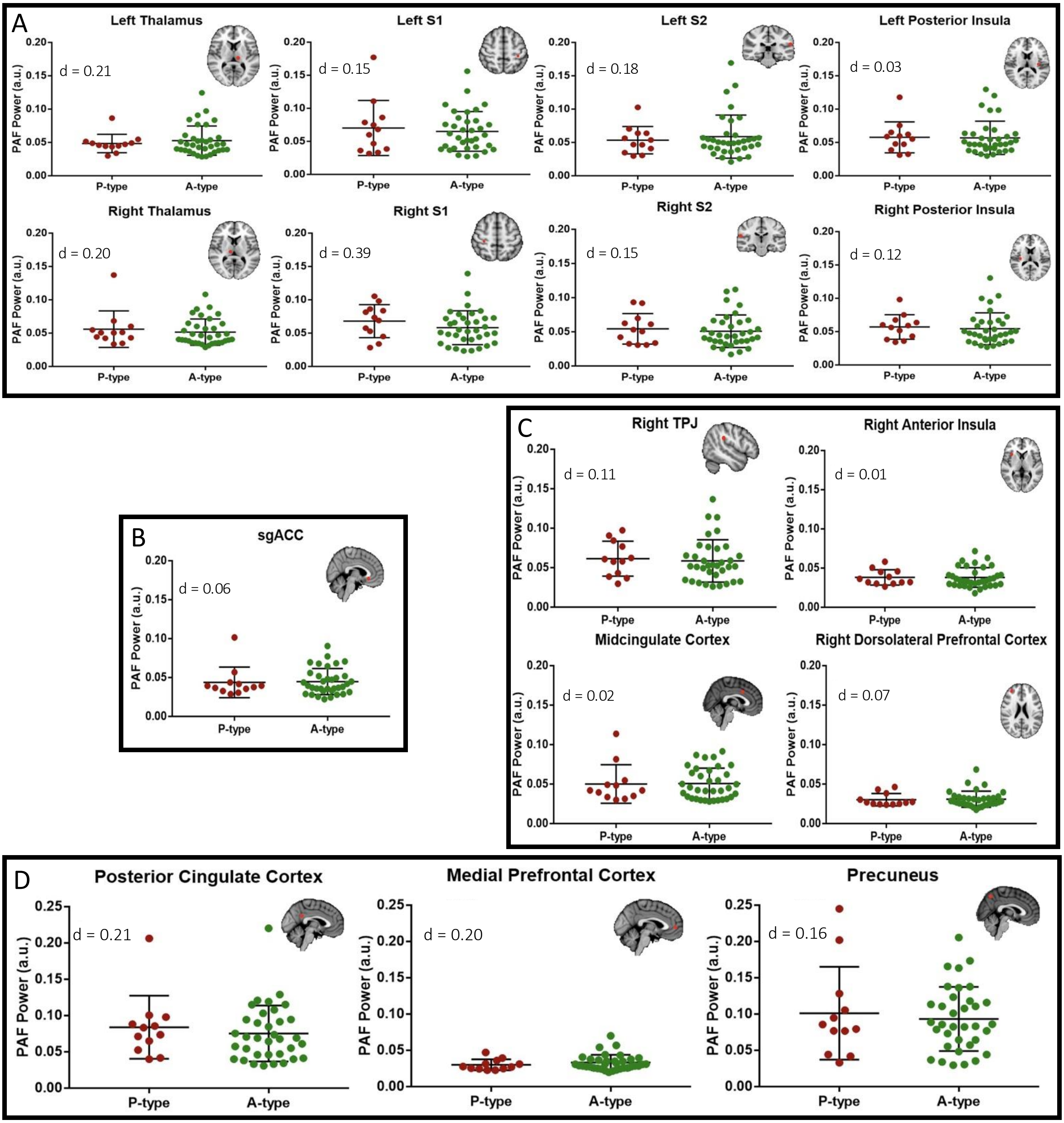

Supplementary Figure 9. Group comparison of power at PAF between P-type (red) and A-type (green)

The mean +/- SD of normalized power at PAF for each group are shown for key regions of interest within the A) ascending nociceptive pathway, B) descending antinociceptive pathway, C) salience network, and D) default mode network. Reported P-values are uncorrected for multiple comparisons. S1, primary somatosensory cortex; S2, secondary somatosensory cortex; sgACC, subgenual anterior cingulate cortex; TPJ, temporoparietal junction.

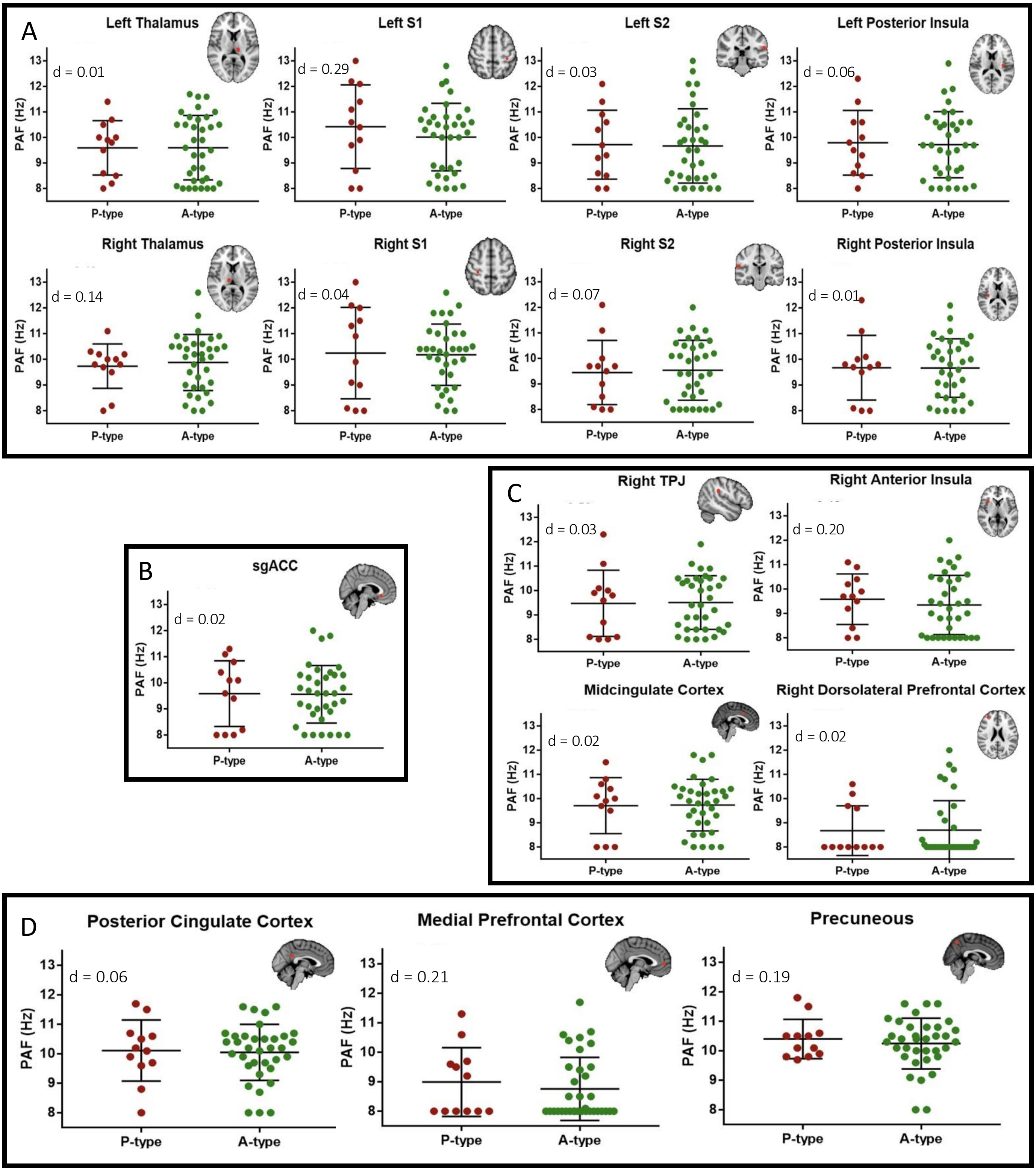

Supplementary Figure 10. Group comparison of PAF between P-type (red) and A-type (green). The mean +/- SD of PAF for each group are shown for key regions of interest within the A) ascending nociceptive pathway, B) descending antinociceptive pathway, C) salience network, and D) default mode network. Reported P-values are uncorrected for multiple comparisons. PAF, peak alpha frequency; S1, primary somatosensory cortex; S2, secondary somatosensory cortex; sgACC, subgenual anterior cingulate cortex; TPJ, temporoparietal junction.

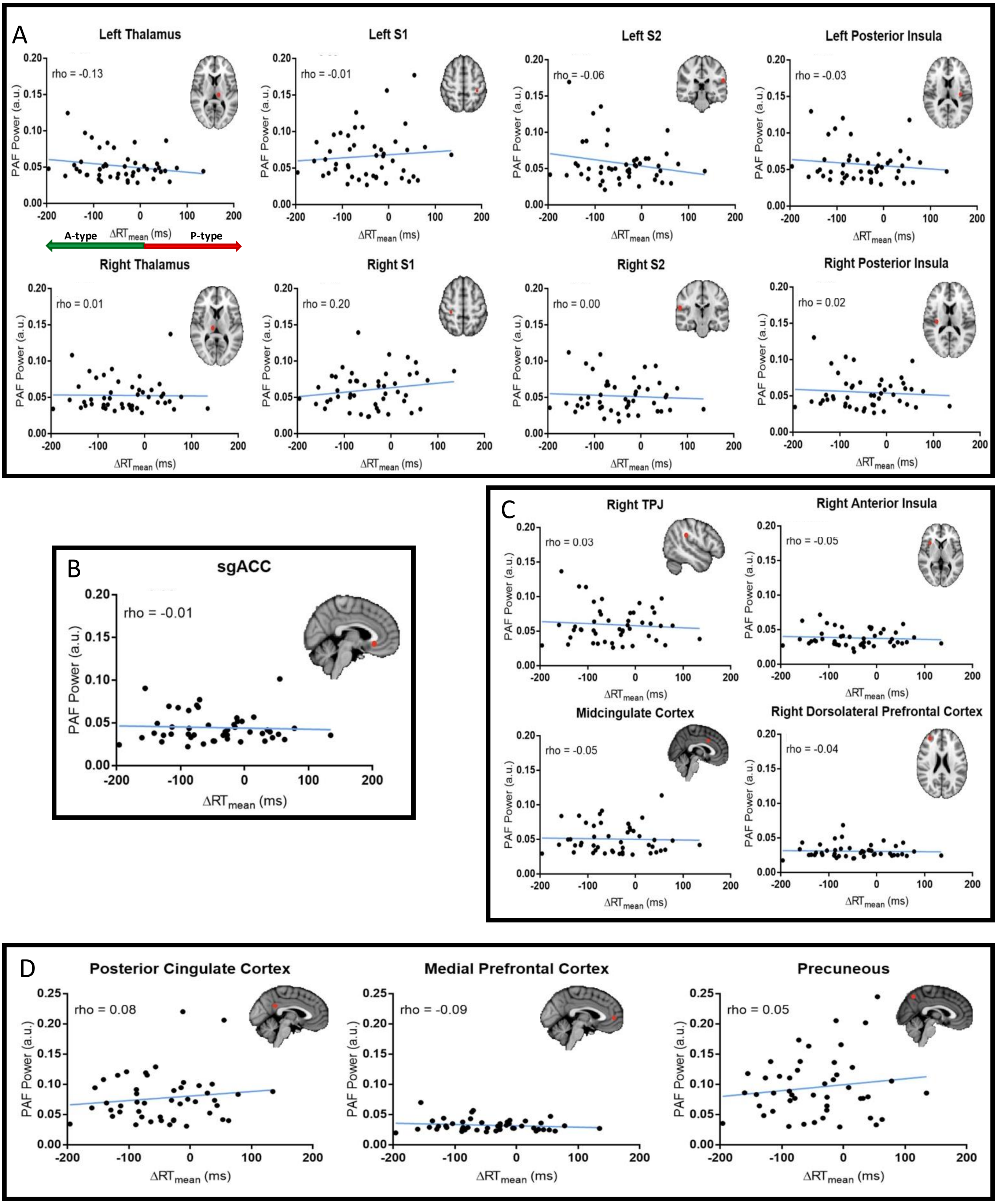

Supplementary Figure 11. Relationship between ΔRTmean and power at PAF. Panels A, B, C, D include ROIs within the ascending nociceptive pathway, descending antinociceptive pathway, salience network, and default mode network, respectively. Reported P-values are uncorrected for multiple comparisons. PAF, peak alpha frequency; S1, primary somatosensory cortex; S2, secondary somatosensory cortex; sgACC, subgenual anterior cingulate cortex; TPJ, temporoparietal junction.

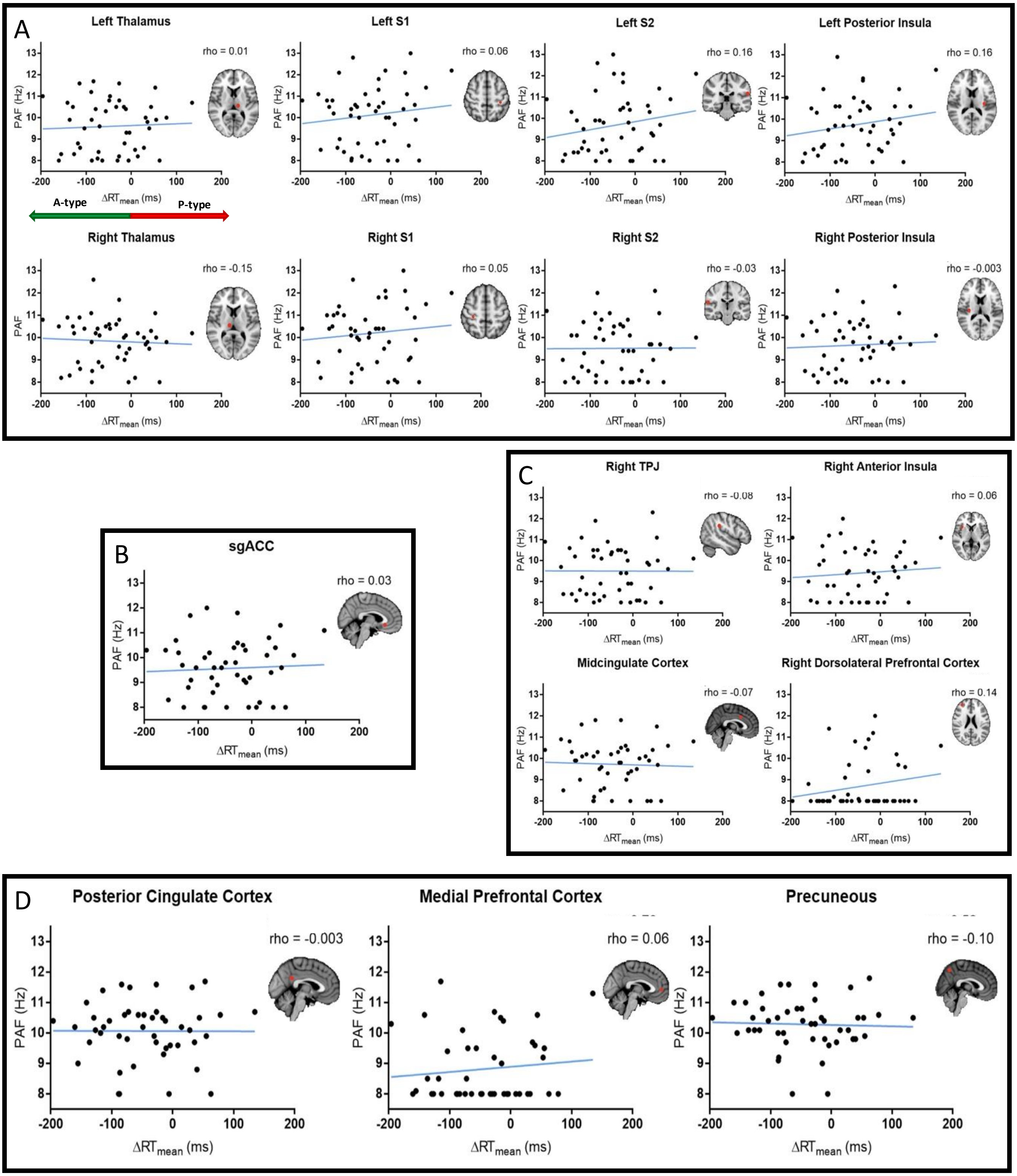

Supplementary Figure 12. Relationship between ΔRTmean and PAF speed. Panels A, B, C, D include ROIs within the ascending nociceptive pathway, descending antinociceptive pathway, salience network, and default mode network, respectively. Reported P-values are uncorrected for multiple comparisons. PAF, peak alpha frequency; S1, primary somatosensory cortex; S2, secondary somatosensory cortex; sgACC, subgenual anterior cingulate cortex; TPJ, temporoparietal junction.

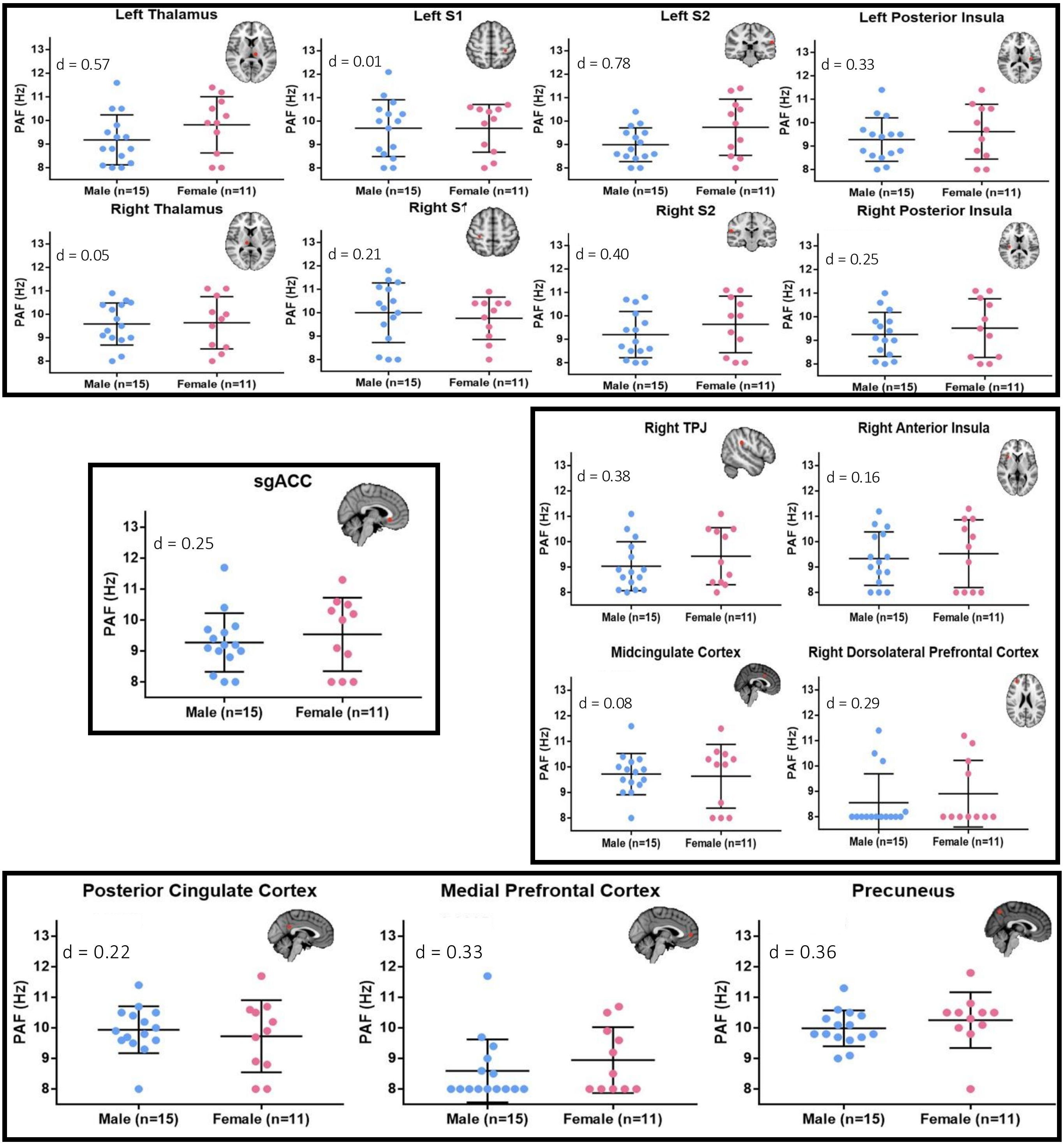

Supplementary Figure 13. **PAF speed difference between male (blue) and female (pink) in the low IAP group**. The mean +/- SD of PAF for each sex are shown for key regions of interest within the A) ascending nociceptive pathway, B) descending antinociceptive pathway, C) salience network, and D) default mode network. IAP, intrinsic attention to pain; PAF, peak alpha frequency; S1, primary somatosensory cortex; S2, secondary somatosensory cortex; sgACC, subgenual anterior cingulate cortex; TPJ, temporoparietal junction.

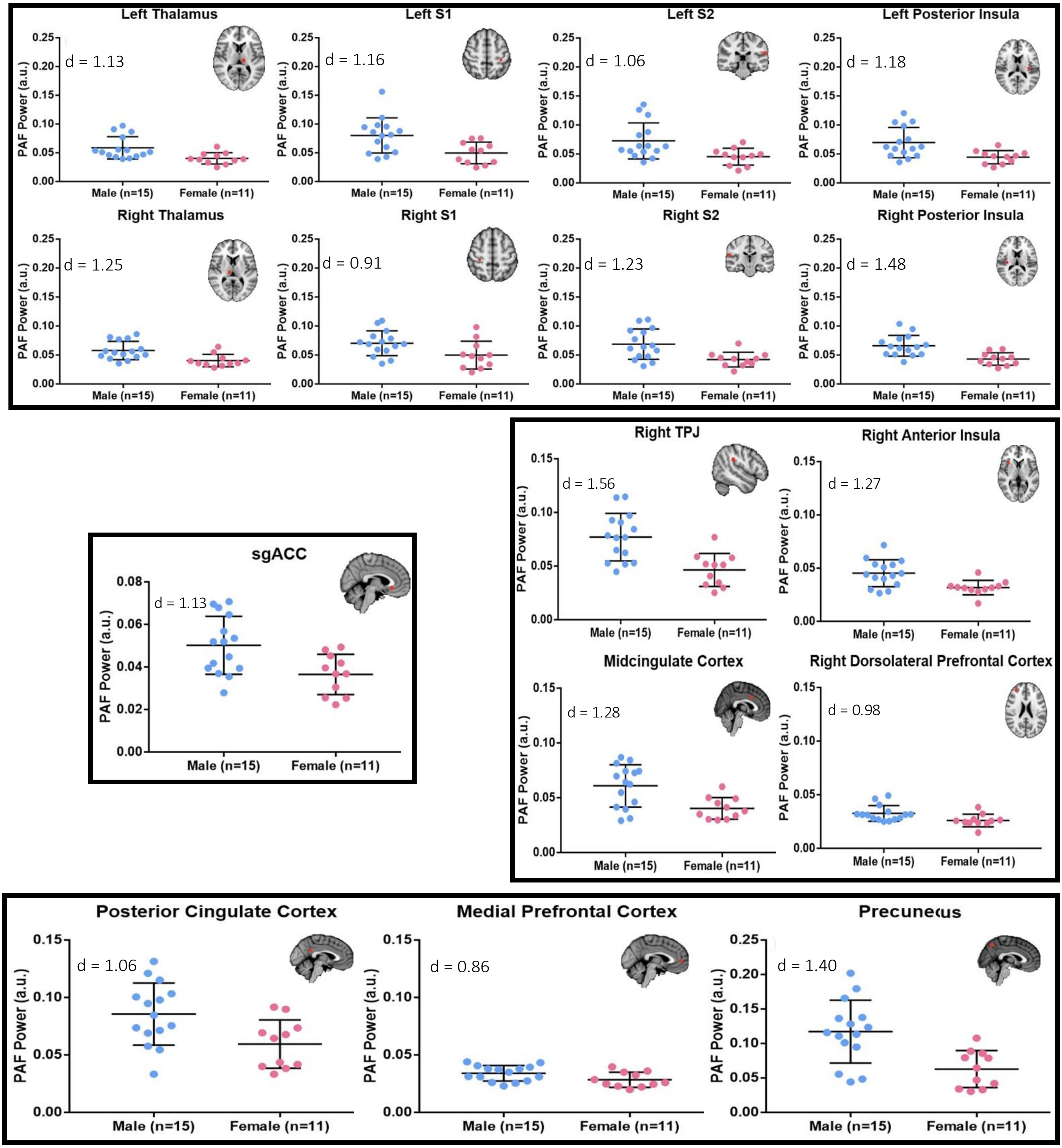

Supplementary Figure 14. **Power at PAF difference between male (blue) and female (pink) in the low IAP group.** The mean +/- SD of power at PAF for each sex are shown for key regions of interest within the A) ascending nociceptive pathway, B) descending antinociceptive pathway, C) salience network, and D) default mode network. IAP, intrinsic attention to pain; PAF, peak alpha frequency; S1, primary somatosensory cortex; S2, secondary somatosensory cortex; sgACC, subgenual anterior cingulate cortex; TPJ, temporoparietal junction.

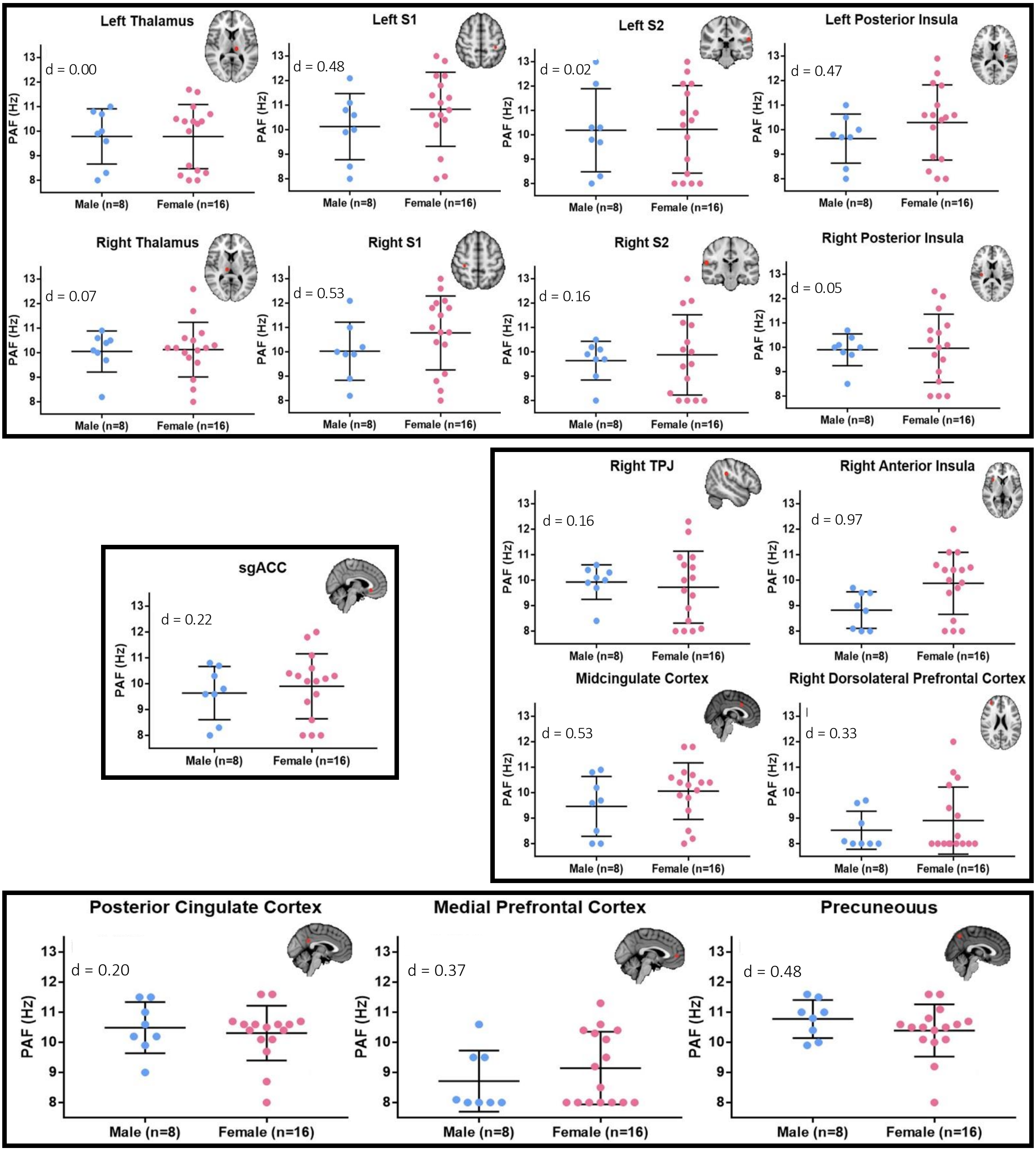

Supplementary Figure 15. PAF Speed difference between male (blue) and female (pink) in the high IAP group.
The mean +/- SD of power at PAF for each sex are shown for key regions of interest within the A) ascending nociceptive pathway, B) descending antinociceptive pathway, C) salience network, and D) default mode network. IAP, intrinsic attention to pain; PAF, peak alpha frequency; S1, primary somatosensory cortex; S2, secondary somatosensory cortex; sgACC, subgenual anterior cingulate cortex; TPJ, temporoparietal junction.

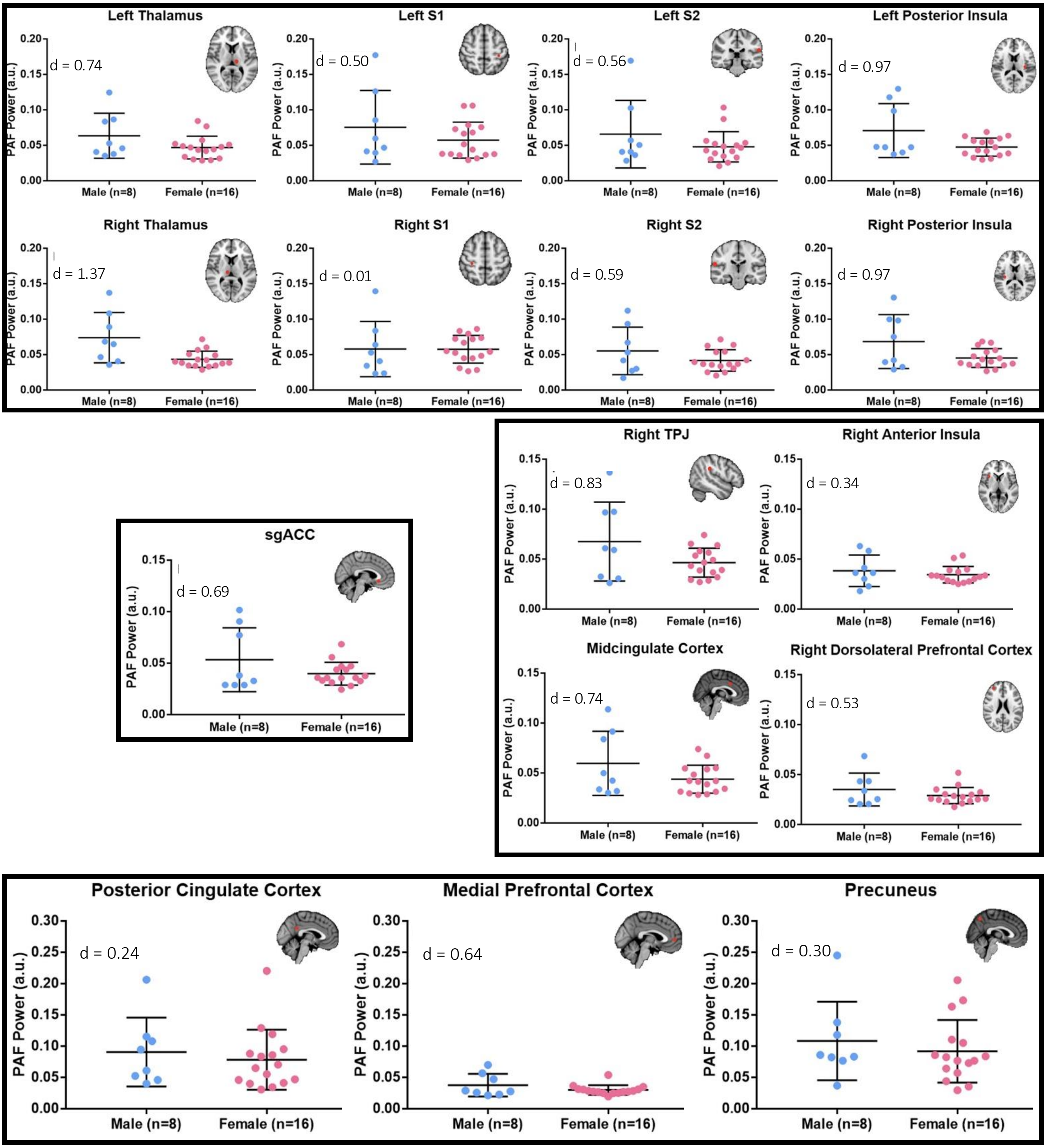

Supplementary Figure 16. **Power at PAF difference between male (blue) and female (pink) in the high IAP group**. The mean +/- SD of power at PAF for each sex are shown for key regions of interest within the A) ascending nociceptive pathway, B) descending antinociceptive pathway, C) salience network, and D) default mode network. IAP, intrinsic attention to pain; PAF, peak alpha frequency; S1, primary somatosensory cortex; S2, secondary somatosensory cortex; sgACC, subgenual anterior cingulate cortex; TPJ, temporoparietal junction.

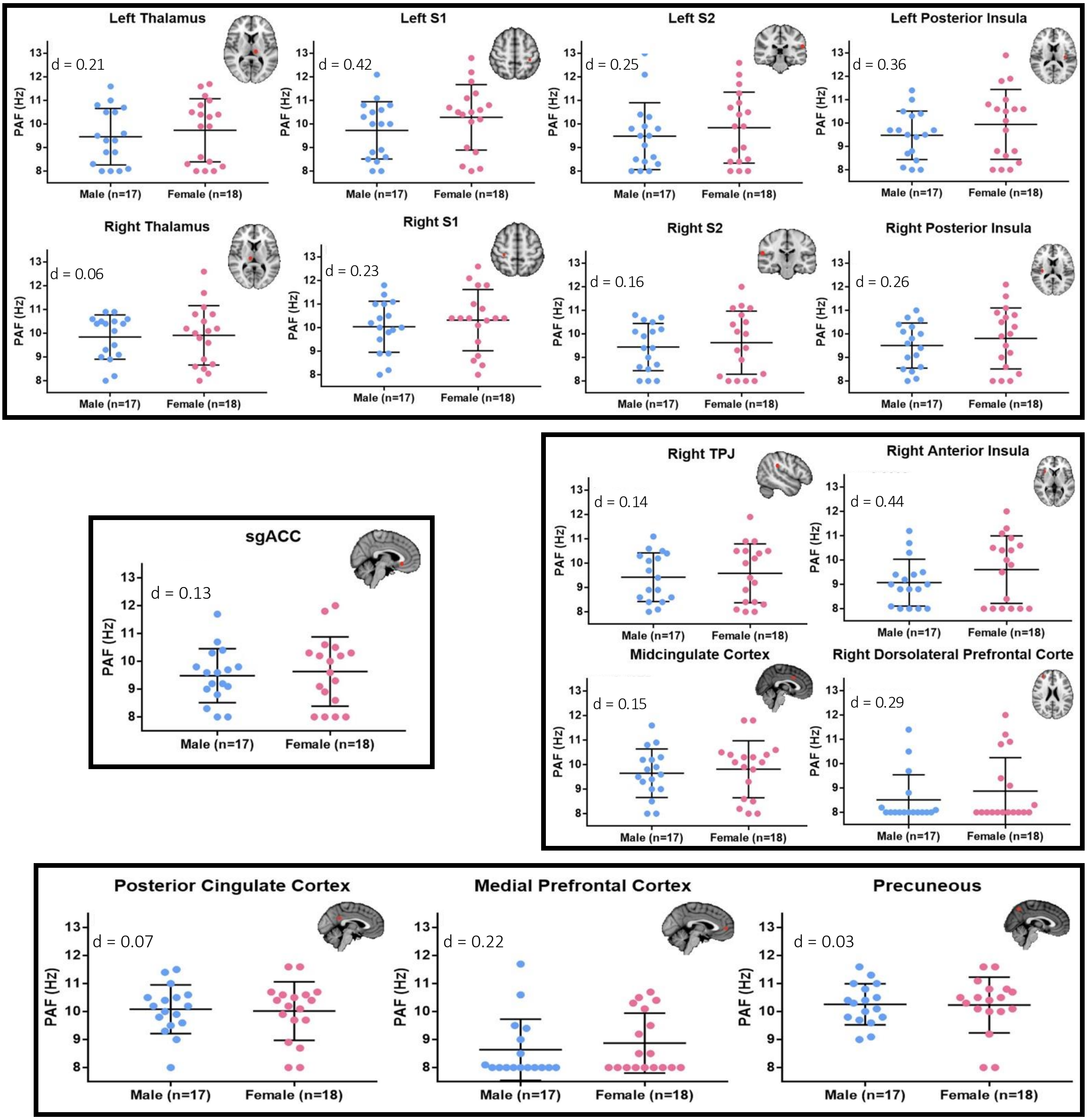

Supplementary Figure 17. **PAF difference between male (blue) and female (pink) within A-type individuals.** Panels A, B, C, D include ROIs within the ascending nociceptive pathway, descending antinociceptive pathway, salience network, and default mode network, respectively. PAF; peak alpha frequency; S1, primary somatosensory cortex; S2, secondary somatosensory cortex; sgACC, subgenual anterior cingulate cortex; TPJ, temporoparietal junction.

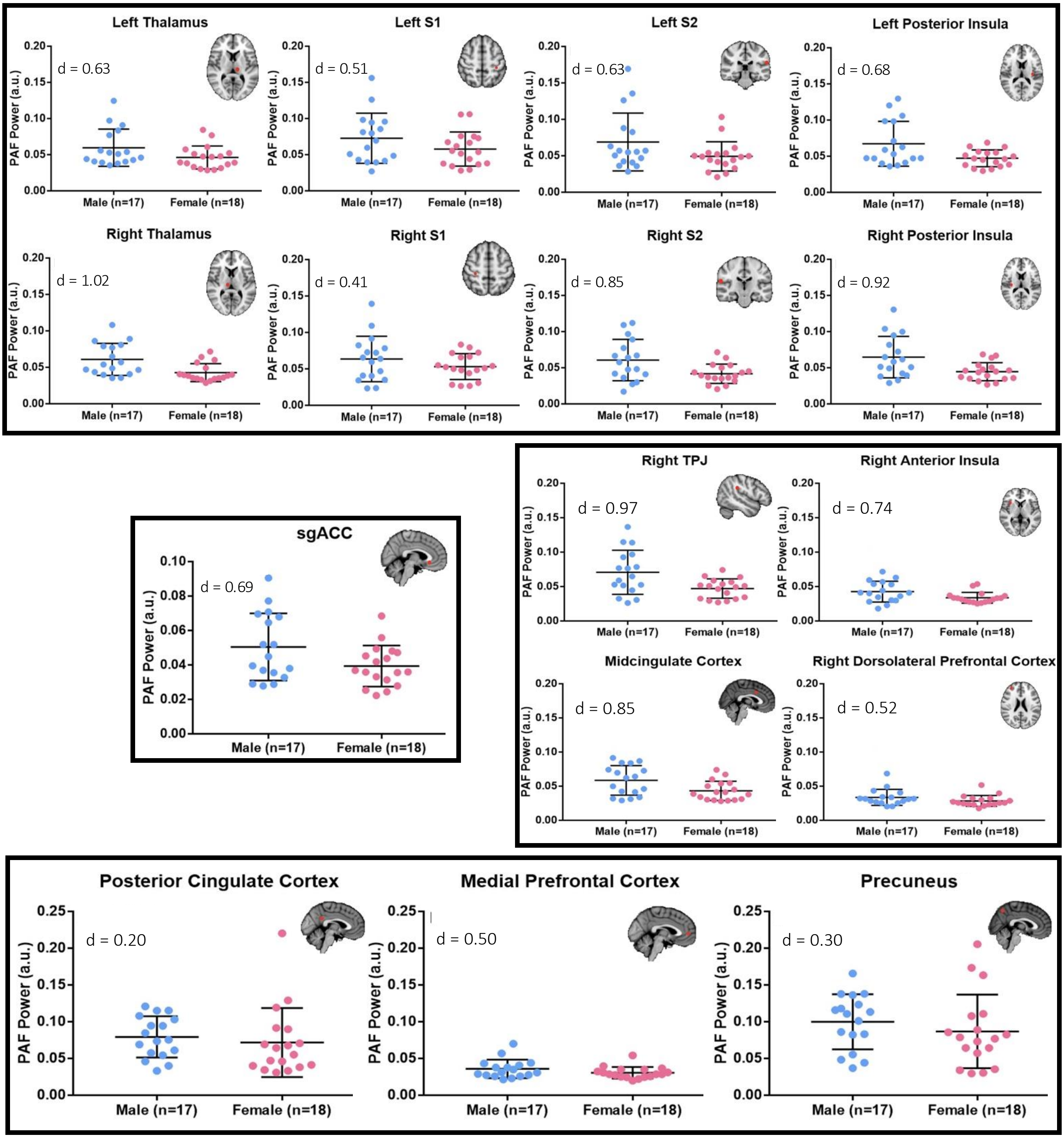

Supplementary Figure 18. **Power at PAF difference between male (blue) and female (pink) within A-type group.** Panels A, B, C, D include ROIs within the ascending nociceptive pathway, descending antinociceptive pathway, salience network, and default mode network, respectively. PAF, peak alpha frequency; S1, primary somatosensory cortex; S2, secondary somatosensory cortex; sgACC, subgenual anterior cingulate cortex; TPJ, temporoparietal junction.

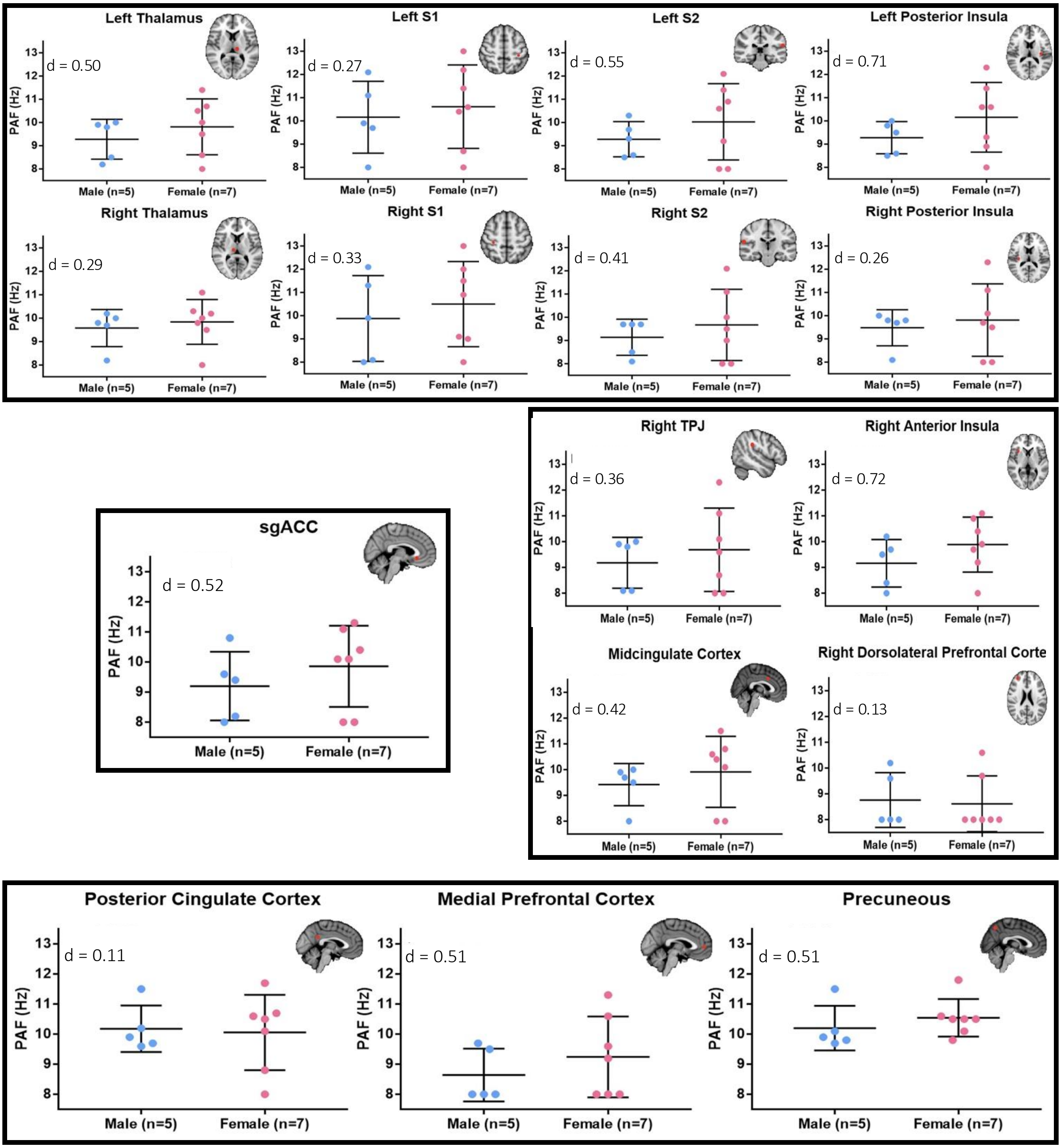

Supplementary Figure 19. **PAF difference between male (blue) and female (pink) within P-type group**. Panels A, B, C, D include ROIs within the ascending nociceptive pathway, descending antinociceptive pathway, salience network, and default mode network, respectively. PAF, peak alpha frequency; S1, primary somatosensory cortex; S2, secondary somatosensory cortex; sgACC, subgenual anterior cingulate cortex; TPJ, temporoparietal junction.

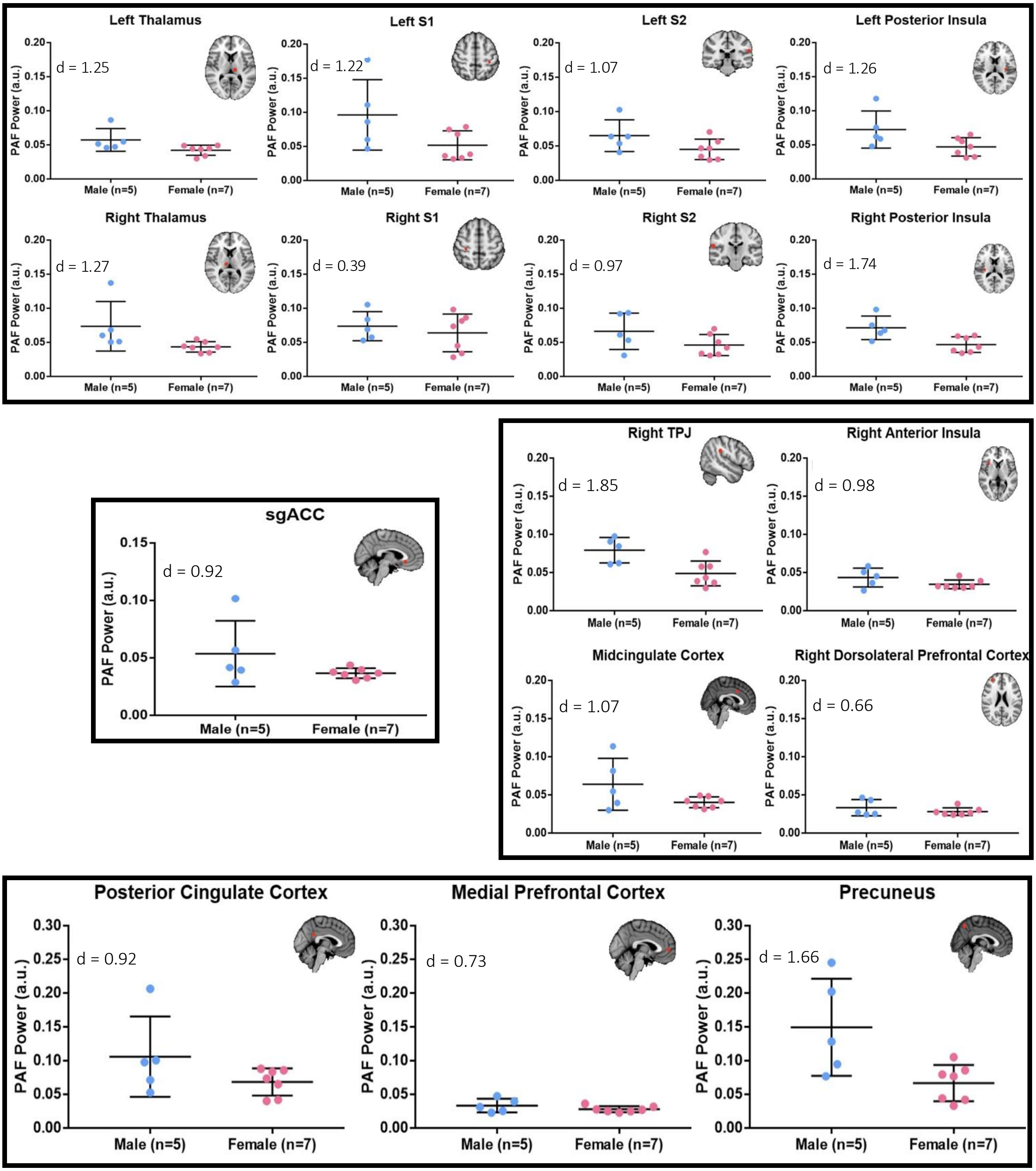

Supplementary Figure 20. **Power at PAF difference between male (blue) and female (pink) within P-type group**. Panels A, B, C, D include ROIs within the ascending nociceptive pathway, descending antinociceptive pathway, salience network, and default mode network, respectively. PAF, peak alpha frequency; S1, primary somatosensory cortex; S2, secondary somatosensory cortex; sgACC, subgenual anterior cingulate cortex; TPJ, temporoparietal junction.
