# Supplementary material for: Alpha and Theta Oscillations Associated With Behavioral Phenotypes of Pain–Attention Interaction
Source: Brain Behav. 2025 Jan 19;15(1):e70190. doi: 10.1002/brb3.70190 (PMC11743985; doi:10.1002/brb3.70190)
Supplement: Supplementary file 2 — Supplementary Tables [file BRB3-15-e70190-s001.pdf]

Supplementary Tables

Alpha and Theta Oscillations associated with Behavioral Phenotypes of Pain-Attention Interaction

Nikou Kelardashti, Benjamin T Dunkley, Rima El-Sayed, Vaidhehi Veena Sanmugananthan, Junseok Andrew Kim, Natalie Rae Osborne, Joshua C. Cheng, Anton Rogachov, Rachael L. Bosma, Ariana E. Besik, and Karen Deborah Davis

Supplementary Table 1. Mean±SD of PAF speed and PAF power of high and low IAP groups and sex-segregated information for the nodes of ascending nociceptive pathway. P-values and effect sizes of group comparisons are included in the tables as well. *P-values which are significant after correcting for multiple comparisons.

Left Thalamus

	High IAP				Low IAP				p-value	Effect size
PAF Speed	9.78±1.22				10.10±0.74				0.30	0.32
PAF Power	0.05±0.02				0.09±0.05				0.84	0.15
	M	F	p-value	Effect size	M	F	p-value	Effect size		
PAF Speed	9.79 ±1.1	9.78 ±1.3	0.91	0.00	9.99 ±0.59	10.254 ±0.91	0.11	0.57		
PAF Power	0.06 ±0.03	0.05 ±0.02	0.26	0.74	0.12 ±0.04	0.06 ±0.03	0.004*	1.13		

Right Thalamus

	High IAP				Low IAP				p-value	Effect size
PAF Speed	10.10±1.01				9.61±0.97				0.13	0.50
PAF Power	0.05±0.03				0.050±0.02				0.85	0.14
	M	F	p-value	Effect size	M	F	p-value	Effect size		
PAF Speed	10.05 ±0.84	10.12 ±1.1	0.92	0.07	9.59 ±0.90	9.64 ±1.12	0.93	0.05		
PAF Power	0.07 ±0.03	0.04 ±0.01	0.01	1.37	0.06 ±0.02	0.04 ±0.01	0.003*	1.25		

Left S1

	High IAP				Low IAP				p-value	Effect size
PAF Speed	10.60±1.47				9.70±1.11				0.01	0.69
PAF Power	0.06±0.04				0.07±0.03				0.37	0.12
	M	F	p-value	Effect size	M	F	p-value	Effect size		
PAF Speed	10.12 ±1.3	10.83 ±1.5	0.20	0.48	9.70 ±1.2	9.69 ±1.0	0.83	0.01		
PAF Power	0.07 ±0.05	0.06 ±0.02	0.45	0.50	0.08 ±0.03	0.05 ±0.02	0.005*	1.16		

Right S1

	High IAP				Low IAP				p-value	Effect size
PAF Speed	10.52±1.44				9.90±1.12				0.11	0.49
PAF Power	0.06±0.03				0.06±0.02				0.56	0.16
	M	F	p-value	Effect size	M	F	p-value	Effect size		
PAF Speed	10.02 ±1.2	10.77 ±1.5	0.19	0.53	10.00 ±1.3	9.76 ±0.9	0.48	0.21		
PAF Power	0.06 ±0.04	0.06 ±0.02	0.53	0.01	0.07 ±0.02	0.05 ±0.02	0.03*	0.91		

Supplementary Table 2. Mean±SD of PAF speed and PAF power of high and low IAP groups and sex-segregated information for the nodes of ascending nociceptive pathway. P-values and effect sizes of group comparisons are included in the tables as well. *P-values which are significant after correcting for multiple comparisons.

Left S2										
	High IAP				Low IAP				p-value	Effect size
PAF Speed	10.21±1.73				9.31±1.00				0.10	0.65
PAF Power	0.05±0.03				0.06±0.04				0.08	0.23
	M	F	p-value	Effect size	M	F	p-value	Effect size		
PAF Speed	10.18 ±1.7	10.22 ±1.8	0.84	0.02	8.99 ±0.7	9.73 ±1.2	0.13	0.78		
PAF Power	0.07 ±0.05	0.05 ±0.02	0.53	0.56	0.07 ±0.03	0.04 ±0.01	0.009*	1.06		

Right S2										
	High IAP				Low IAP				p-value	Effect size
PAF Speed	9.80±1.40				9.38±1.09				0.41	0.33
PAF Power	0.05±0.02				0.06±0.02				0.08	0.46
	M	F	p-value	Effect size	M	F	p-value	Effect size		
PAF Speed	9.64 ±0.80	9.87 ±1.6	0.94	0.16	9.20 ±1.0	9.64 ±1.2	0.39	0.40		
PAF Power	0.05 ±0.03	0.04 ±0.01	0.57	0.59	0.07 ±0.03	0.04 ±0.01	0.009*	1.23		

Left Posterior Insula										
	High IAP				Low IAP				p-value	Effect size
PAF Speed	10.07±1.39				9.42±1.03				0.08	0.54
PAF Power	0.05±0.03				0.06±0.024				0.48	0.15
	M	F	p-value	Effect size	M	F	p-value	Effect size		
PAF Speed	9.63 ±1.0	10.29 ±1.5	0.21	0.47	9.28 ±0.9	9.61 ±1.2	0.45	0.33		
PAF Power	0.07 ±0.04	0.05 ±0.01	0.21	0.97	0.07 ±0.03	0.04 ±0.01	0.004*	1.18		

Right Posterior Insula										
	High IAP				Low IAP				p-value	Effect size
PAF Speed	9.94±1.18				9.36±1.06				0.11	0.51
PAF Power	0.05±0.03				0.06±0.02				0.22	0.15
	M	F	p-value	Effect size	M	F	p-value	Effect size		
PAF Speed	9.90 ±0.6	10.00 ±1.4	0.89	0.05	9.25 ±0.9	9.52 ±1.2	0.60	0.25		
PAF Power	0.07 ±0.04	0.04 ±0.01	0.26	0.97	0.07 ±0.02	0.04 ±0.01	0.0004*	1.48		

Supplementary Table 3. Mean±SD of PAF speed and PAF power of high and low IAP groups and sex-segregated information for the nodes of salience network. P-values and effect sizes of group comparisons are included in the tables as well. *P-values which are significant after correcting for multiple comparisons.

Right Temporoparietal Junction										
	High IAP				Low IAP				p-value	Effect size
PAF Speed	9.79±1.20				9.20±1.03				0.15	0.53
PAF Power	0.05±0.03				0.06±0.02				0.09	0.41
	M	F	p-value	Effect size	M	F	p-value	Effect size		
PAF Speed	9.92 ±0.7	9.72 ±1.4	0.73	0.16	9.03 ±1.0	9.43 ±1.1	0.44	0.38		
PAF Power	0.07 ±0.04	0.05 ±0.01	0.32	0.83	0.08 ±0.02	0.05 ±0.01	0.0006*	1.56		

Right Anterior Insula										
	High IAP				Low IAP				p-value	Effect size
PAF Speed	9.52±1.17				9.42±1.16				0.74	0.09
PAF Power	0.04±0.01				0.04±0.01				0.29	0.32
	M	F	p-value	Effect size	M	F	p-value	Effect size		
PAF Speed	8.82 ±0.7	9.87 ±1.2	0.03	0.97	9.33 ±1.0	9.53 ±1.3	0.75	0.16		
PAF Power	0.04 ±0.16	0.03 ±0.01	0.57	0.34	0.04 ±0.01	0.03 ±0.01	0.01*	1.27		

Midcingulate Cortex										
	High IAP				Low IAP				p-value	Effect size
PAF Speed	9.86±1.14				9.69±0.99				0.35	0.17
PAF Power	0.05±0.02				0.05±0.02				0.41	0.15
	M	F	p-value	Effect size	M	F	p-value	Effect size		
PAF Speed	9.46 ±1.2	10.06 ±1.1	0.29	0.53	9.72 ±0.8	9.64 ±1.2	0.65	0.08		
PAF Power	0.06 ±0.03	0.04 ±0.01	0.29	0.74	0.06 ±0.02	0.04 ±0.01	0.008*	1.28		

Right Dorsolateral Prefrontal Cortex										
	High IAP				Low IAP				p-value	Effect size
PAF Speed	8.78±1.15				8.70±1.20				0.49	0.06
PAF Power	0.03±0.01				0.03±0.01				0.66	0.11
	M	F	p-value	Effect size	M	F	p-value	Effect size		
PAF Speed	8.52 ±0.7	8.91 ±1.3	0.87	0.33	8.55 ±1.1	8.91 ±1.3	0.58	0.29		
PAF Power	0.03 ±0.02	0.03 ±0.01	0.70	0.53	0.03 ±0.01	0.03 ±0.01	0.009*	0.98		

Supplementary Table 4. Mean±SD of PAF speed and PAF power of high and low IAP groups and sex-segregated information for the nodes of default mode network and descending nociceptive pathway. P-values and effect sizes of group comparisons are included in the tables as well. *P-values which are significant after correcting for multiple comparisons.

Posterior Cingulate Cortex										
	High IAP				Low IAP				p-value	Effect size
PAF Speed	10.37±0.88				9.85±0.95				0.02	0.56
PAF Power	0.08±0.05				0.07±0.03				0.99	0.20
	M	F	p-value	Effect size	M	F	p-value	Effect size		
PAF Speed	10.49 ±0.8	10.30 ±0.9	0.87	0.20	9.94 ±0.8	9.73 ±1.2	0.87	0.22		
PAF Power	0.09 ±0.05	0.08 ±0.05	0.65	0.24	0.08 ±0.03	0.06 ±0.02	0.01*	1.06		

Medial Prefrontal Cortex										
	High IAP				Low IAP				p-value	Effect size
PAF Speed	9.00±1.14				8.74±1.04				0.44	0.23
PAF Power	0.03±0.01				0.03±0.01				0.57	0.10
	M	F	p-value	Effect size	M	F	p-value	Effect size		
PAF Speed	8.71 ±1.0	9.14 ±1.2	0.56	0.37	8.59 ±1.0	8.94 ±1.1	0.37	0.33		
PAF Power	0.04 ±0.02	0.03 ±0.01	0.70	0.64	0.03 ±0.01	0.03 ±0.01	0.04*	0.86		

Precuneus										
	High IAP				Low IAP				p-value	Effect size
PAF Speed	10.52±0.80				10.10±0.74				0.01	0.55
PAF Power	0.10±0.05				0.09±0.05				0.84	0.06
	M	F	p-value	Effect size	M	F	p-value	Effect size		
PAF Speed	10.77 ±0.6	10.39 ±0.9	0.42	0.48	9.99 ±0.6	10.25 ±0.9	0.11	0.36		
PAF Power	0.11 ±0.06	0.09 ±0.05	0.35	0.30	0.12 ±0.04	0.06 ±0.03	0.0008*	1.40		

Subgenual Anterior Cingulate Cortex										
	High IAP				Low IAP				p-value	Effect size
PAF Speed	9.81±1.17				9.38±1.04				0.14	0.39
PAF Power	0.04±0.02				0.04±0.01				0.34	0.01
	M	F	p-value	Effect size	M	F	p-value	Effect size		
PAF Speed	9.64 ±1.0	9.90 ±1.2	0.71	0.22	9.27 ±0.9	9.54 ±1.2	0.55	0.25		
PAF Power	0.05 ±0.03	0.04 ±0.01	0.83	0.69	0.05 ±0.01	0.04 ±0.01	0.002*	1.13		

Supplementary Table 5. Mean±SD of PAF speed and PAF power of P- and A-type groups and sex-segregated information for the nodes of ascending nociceptive pathway. P-values and effect sizes of group comparisons are included in the tables as well.

Left Thalamus										
	P-type				A-type				p-value	Effect size
PAF Speed	9.59±1.06				9.60±1.26				0.99	0.01
PAF Power	0.05±0.01				0.05±0.02				0.95	0.21
	M	F	p-value	Effect size	M	F	p-value	Effect size		
PAF Speed	9.28 ±0.9	9.81 ±1.2	0.37	0.50	9.46 ±1.2	9.73 ±1.3	0.59	0.21		
PAF Power	0.06 ±0.02	0.04 ±0.01	0.03	1.25	0.06 ±0.03	0.05 ±0.02	0.08	0.63		

Right Thalamus										
	P-type				A-type				p-value	Effect size
PAF Speed	9.73±0.86				9.88±1.09				0.48	0.14
PAF Power	0.056±0.027				0.05±0.02				0.50	0.20
	M	F	p-value	Effect size	M	F	p-value	Effect size		
PAF Speed	9.58 ±0.8	9.84 ±0.9	0.56	0.29	9.84 ±0.9	9.91 ±1.2	0.89	0.06		
PAF Power	0.07 ±0.04	0.04 ±0.01	0.02	1.27	0.06 ±0.02	0.04 ±0.01	0.002*	1.02		

Left S1										
	P-type				A-type				p-value	Effect size
PAF Speed	10.42±1.64				10.01±1.32				0.51	0.29
PAF Power	0.07±0.04				0.06±0.30				0.97	0.15
	M	F	p-value	Effect size	M	F	p-value	Effect size		
PAF Speed	10.16 ±1.5	10.61 ±1.8	0.56	0.27	9.73 ±1.2	10.28 ±1.4	0.20	0.42		
PAF Power	0.10 ±0.05	0.05 ±0.02	0.07	1.22	0.07 ±0.03	0.06 ±0.02	0.19	0.51		

Right S1										
	P-type				A-type				p-value	Effect size
PAF Speed	10.24±1.78				10.18±1.19				0.87	0.04
PAF Power	0.07±0.02				0.06±0.02				0.15	0.39
	M	F	p-value	Effect size	M	F	p-value	Effect size		
PAF Speed	9.88 ±1.8	10.50 ±1.8	0.50	0.33	10.03 ±1.1	10.32 ±1.3	0.50	0.23		
PAF Power	0.07 ±0.02	0.06 ±0.03	0.6	0.39	0.06 ±0.03	0.05 ±0.02	0.48	0.41		

Supplementary Table 6. Mean±SD of PAF speed and PAF power of P- and A-type groups and sex-segregated information for the nodes of ascending nociceptive pathway. P-values and effect sizes of group comparisons are included in the tables as well.

Left S2										
	P-type				A-type				p-value	Effect size
PAF Speed	9.72±1.35				9.67±1.45				0.80	0.03
PAF Power	0.05±0.02				0.06±0.03				0.97	0.18
	M	F	p-value	Effect size	M	F	p-value	Effect size		
PAF Speed	9.28 ±0.7	10.03 ±1.6	0.67	0.55	9.48 ±1.4	9.84 ±1.5	0.40	0.25		
PAF Power	0.06 ±0.02	0.04 ±0.01	0.15	1.07	0.07 ±0.04	0.05 ±0.02	0.09	0.63		

Right S2										
	P-type				A-type				p-value	Effect size
PAF Speed	9.45±1.26				9.54±1.18				0.73	0.07
PAF Power	0.05±0.02				0.05±0.02				0.66	0.15
	M	F	p-value	Effect size	M	F	p-value	Effect size		
PAF Speed	9.14 ±0.8	9.67 ±1.5	0.83	0.41	9.44 ±1.0	9.63 ±1.3	0.70	0.16		
PAF Power	0.07 ±0.03	0.05 ±0.01	0.27	0.97	0.06 ±0.03	0.04 ±0.01	0.04	0.85		

Left Posterior Insula										
	P-type				A-type				p-value	Effect size
PAF Speed	9.79±1.27				9.72±1.30				0.84	0.06
PAF Power	0.06±0.02				0.06±0.02				0.57	0.03
	M	F	p-value	Effect size	M	F	p-value	Effect size		
PAF Speed	9.28 ±0.7	10.16 ±1.4	0.32	0.71	9.48 ±1.0	9.94 ±1.5	0.30	0.36		
PAF Power	0.07 ±0.02	0.05 ±0.01	0.07	1.26	0.07 ±0.03	0.05 ±0.01	0.06	0.68		

Right Posterior Insula										
	P-type				A-type				p-value	Effect size
PAF Speed	9.67±1.26				9.66±1.14				0.85	0.01
PAF Power	0.057±0.02				0.05±0.02				0.43	0.12
	M	F	p-value	Effect size	M	F	p-value	Effect size		
PAF Speed	9.48 ±0.8	9.81 ±1.6	0.99	0.26	9.50 ±0.9	9.81 ±1.3	0.50	0.26		
PAF Power	0.07 ±0.02	0.05 ±0.01	0.02	1.74	0.06 ±0.03	0.04 ±0.01	0.02	0.92		

Supplementary Table 7. Mean±SD of PAF speed and PAF power of P- and A-type groups and sex-segregated information for the nodes of the salience network. P-values and effect sizes of group comparisons are included in the tables as well.

**Right Temporoparietal Junction**										
	**P-type**				**A-type**				**p-value**	**Effect size**
**PAF Speed**	9.47±1.36				9.51±1.10				0.59	0.03
**PAF Power**	0.06±0.02				0.06±0.03				0.46	0.11
	**M**	**F**	**p-value**	**Effect size**	**M**	**F**	**p-value**	**Effect size**		
**PAF Speed**	9.18 ±1.0	9.69 ±1.6	0.85	0.36	9.42 ±1.0	9.58 ±1.2	0.79	0.14		
**PAF Power**	0.08 ±0.02	0.05 ±0.02	0.01	1.85	0.07 ±0.03	0.05 ±0.01	0.02	0.97		

**Right Anterior Insula**										
	**P-type**				**A-type**				**p-value**	**Effect size**
**PAF Speed**	9.58±1.03				9.35±1.21				0.48	0.20
**PAF Power**	0.04±0.01				0.04±0.01				0.76	0.01
	**M**	**F**	**p-value**	**Effect size**	**M**	**F**	**p-value**	**Effect size**		
**PAF Speed**	9.16 ±0.9	9.89 ±1.1	0.25	0.72	9.07 ±1.0	9.61 ±1.3	0.30	0.44		
**PAF Power**	0.04 ±0.01	0.03 ±0.01	0.27	0.98	0.04 ±0.01	0.03 ±0.01	0.04	0.74		

**Midcingulate Cortex**										
	**P-type**				**A-type**				**p-value**	**Effect size**
**PAF Speed**	9.71±1.16				9.73±1.07				0.99	0.02
**PAF Power**	0.05±0.02				0.05±0.02				0.93	0.02
	**M**	**F**	**p-value**	**Effect size**	**M**	**F**	**p-value**	**Effect size**		
**PAF Speed**	9.42 ±0.8	9.91 ±1.4	0.20	0.42	9.65 ±1.0	9.81 ±1.2	0.50	0.15		
**PAF Power**	0.06 ±0.03	0.04 ±0.01	0.34	1.07	0.06 ±0.02	0.04 ±0.01	0.02	0.85		

**Right Dorsolateral Prefrontal Cortex**										
	**P-type**				**A-type**				**p-value**	**Effect size**
**PAF Speed**	8.67±1.03				8.70±1.21				0.86	0.02
**PAF Power**	0.03±0.01				0.03±0.01				0.82	0.07
	**M**	**F**	**p-value**	**Effect size**	**M**	**F**	**p-value**	**Effect size**		
**PAF Speed**	8.76 ±1.1	8.61 ±1.1	0.93	0.13	8.51 ±1.0	8.87 ±1.4	0.61	0.29		
**PAF Power**	0.03 ±0.01	0.03 ±0.01	0.53	0.66	0.03 ±0.01	0.03 ±0.01	0.13	0.52		

Supplementary Table 8. Mean±SD of PAF speed and PAF power of P- and A-type groups and sex-segregated information for the nodes of default mode network and descending antinociceptive pathway. P-values and effect sizes of group comparisons are included in the tables as well.

Posterior Cingulate Cortex										
	P-type				A-type				p-value	Effect size
PAF Speed	10.11±1.04				10.05±0.95				0.88	0.06
PAF Power	0.08±0.04				0.07±0.04				0.54	0.21
	M	F	p-value	Effect size	M	F	p-value	Effect size		
PAF Speed	10.18 ±0.8	10.06 ±1.2	0.75	0.11	10.08 ±0.9	10.02 ±1.0	0.86	0.07		
PAF Power	0.11 ±0.06	0.07 ±0.02	0.20	0.92	0.08 ±0.03	0.07 ±0.05	0.19	0.20		

Medial Prefrontal Cortex										
	P-type				A-type				p-value	Effect size
PAF Speed	8.99±1.17				8.76±1.07				0.59	0.21
PAF Power	0.03±0.01				0.03±0.01				0.36	0.20
	M	F	p-value	Effect size	M	F	p-value	Effect size		
PAF Speed	8.64 ±0.9	9.24 ±1.3	0.53	0.51	8.63 ±1.1	8.87 ±1.1	0.51	0.22		
PAF Power	0.03 ±0.01	0.03 ±0.00	0.43	0.73	0.04 ±0.01	0.03 ±0.01	0.18	0.50		

Precuneus										
	P-type				A-type				p-value	Effect size
PAF Speed	10.40±0.67				10.25±0.86				0.90	0.19
PAF Power	0.10±0.06				0.09±0.04				0.93	0.16
	M	F	p-value	Effect size	M	F	p-value	Effect size		
PAF Speed	10.20 ±0.7	10.54 ±0.6	0.18	0.51	10.26 ±0.7	10.23 ±1.0	0.63	0.03		
PAF Power	0.15 ±0.07	0.07 ±0.03	0.03	1.66	0.10 ±0.04	0.09 ±0.05	0.14	0.30		

Subgenual Anterior Cingulate Cortex										
	P-type				A-type				p-value	Effect size
PAF Speed	9.58±1.26				9.56±1.10				0.83	0.02
PAF Power	0.04±0.02				0.04±0.02				0.74	0.06
	M	F	p-value	Effect size	M	F	p-value	Effect size		
PAF Speed	9.20 ±1.1	9.86 ±1.3	0.40	0.52	9.48 ±0.97	9.63 ±1.24	0.74	0.13		
PAF Power	0.05 ±0.03	0.04 ±0.00	0.27	0.92	0.05 ±0.02	0.04 ±0.01	0.11	0.69		
